# Supplementary material for: Heterochromatin de novo formation and maintenance in Plasmodium falciparum
Source: PLoS Pathog. 2025 Jun 2;21(6):e1013137. doi: 10.1371/journal.ppat.1013137 (PMC12129197; doi:10.1371/journal.ppat.1013137)
Supplement: S1 Fig — (PDF) [file ppat.1013137.s001.pdf]

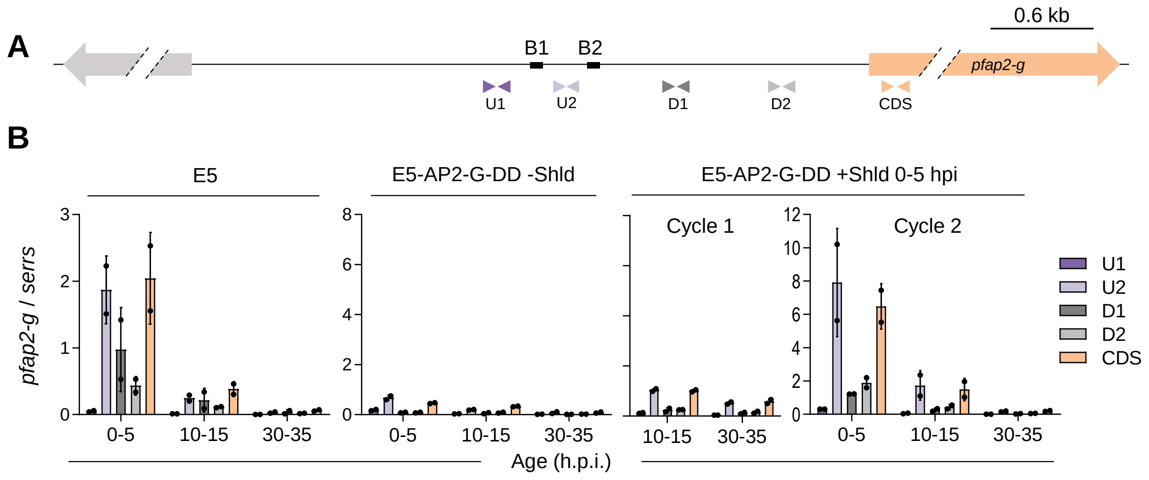

S1 Fig. RT-qPCR validation of the *pfap2-g* TSSs

(A) Schematic of the position of the primers (colored arrowheads) used for validation of the TSSs identified. The position of TSSs block 1 (B1) and block 2 (B2) is also shown.

(B) Validation of the TSSs by RT-qPCR with samples from tightly synchronized E5 and E5-AP2-G-DD cultures, the latter prepared either in the absence (-Shld) or presence (+Shld) of Shld1 and analyzed at the cycle of stabilization (Cycle 1) or at the next cycle (Cycle 2). In cultures with Shld1, the compound was added at 0-5 h post-invasion (hpi) of Cycle 1. Transcript levels are normalized against serine-tRNA ligase (*serrs*). Values are the average of two biological replicates, with s.d.
