# Supplementary material for: Heterochromatin de novo formation and maintenance in Plasmodium falciparum
Source: PLoS Pathog. 2025 Jun 2;21(6):e1013137. doi: 10.1371/journal.ppat.1013137 (PMC12129197; doi:10.1371/journal.ppat.1013137)
Supplement: S2 Fig — (PDF) [file ppat.1013137.s002.pdf]

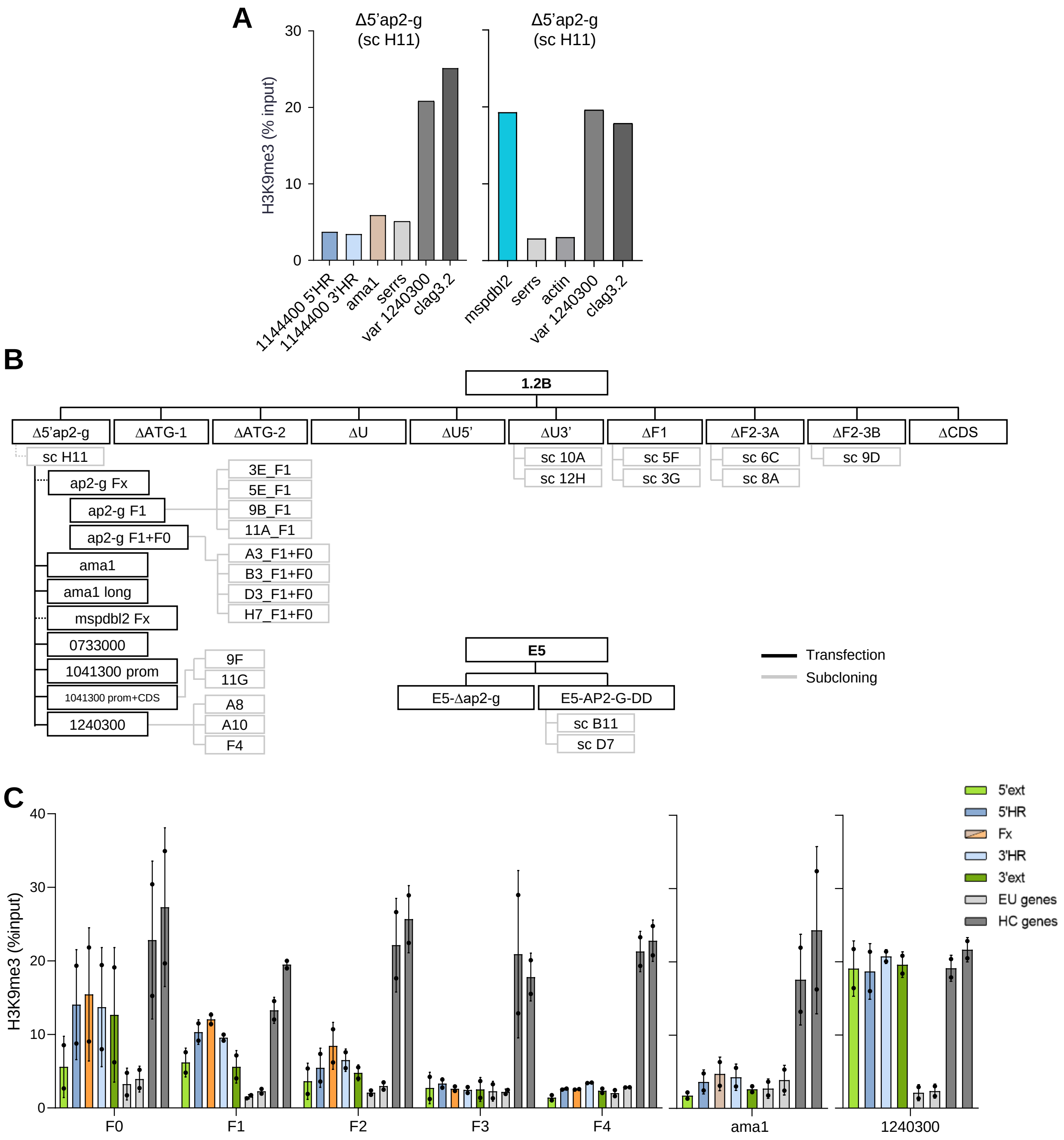

S2 Fig. Parasite lines used in this study and H3K9me3 ChIP-qPCR recovery (% input) values

(A) Validation of the euchromatic or heterochromatic state of the genes studied and ChIP-qPCR controls in the Δ5'ap2-g line (subclone H11). Values are H3K9me3 ChIP-qPCR coverage, calculated as the % DNA recovered in the H3K9me3 IP relative to the input. The left and right panel are two separate ChIP-qPCR experiments in which different genes were analyzed (although *serrs,* the *var* gene and *clag3.2* were analyzed in both experiments). The genes analyzed were the PF3D7_1144400 gene (1144400 5'HR and 3'HR) used for fragment integration, euchromatic genes *ama1* (PF3D7_1133400), serine-tRNA ligase (*serrs*; PF3D7_0717700) and actin I (*actI*; PF3D7_1246200), and heterochromatic genes *var* 1240300 (PF3D7_1240300), *clag3.2*

(PF3D7_0302200) and *mspdbl2* (PF3D7_1036300). In the HC nucleation and maintenance ChIP-qPCR experiments, the genes *serrs* and *actI* were used as ChIP-qPCR negative controls, whereas the genes *var* 1240300 (PF3D7_1240300), *clag3.2* and *mspdbl2* were used as ChIP-qPCR positive controls.

(B) Schematic of the parasite lines used in this study. Fx refers to multiple parasite lines with different fragments integrated (only those for which subclones were generated are shown).

(C) Raw recovery values (% input) for the HC nucleation experiments presented in main Fig 2C (in Fig 2C, results are presented as recovery relative to positive controls). Values are the average and s.d. of two biological replicates.
