# Supplementary material for: Heterochromatin de novo formation and maintenance in Plasmodium falciparum
Source: PLoS Pathog. 2025 Jun 2;21(6):e1013137. doi: 10.1371/journal.ppat.1013137 (PMC12129197; doi:10.1371/journal.ppat.1013137)
Supplement: S3 Fig — (PDF) [file ppat.1013137.s003.pdf]

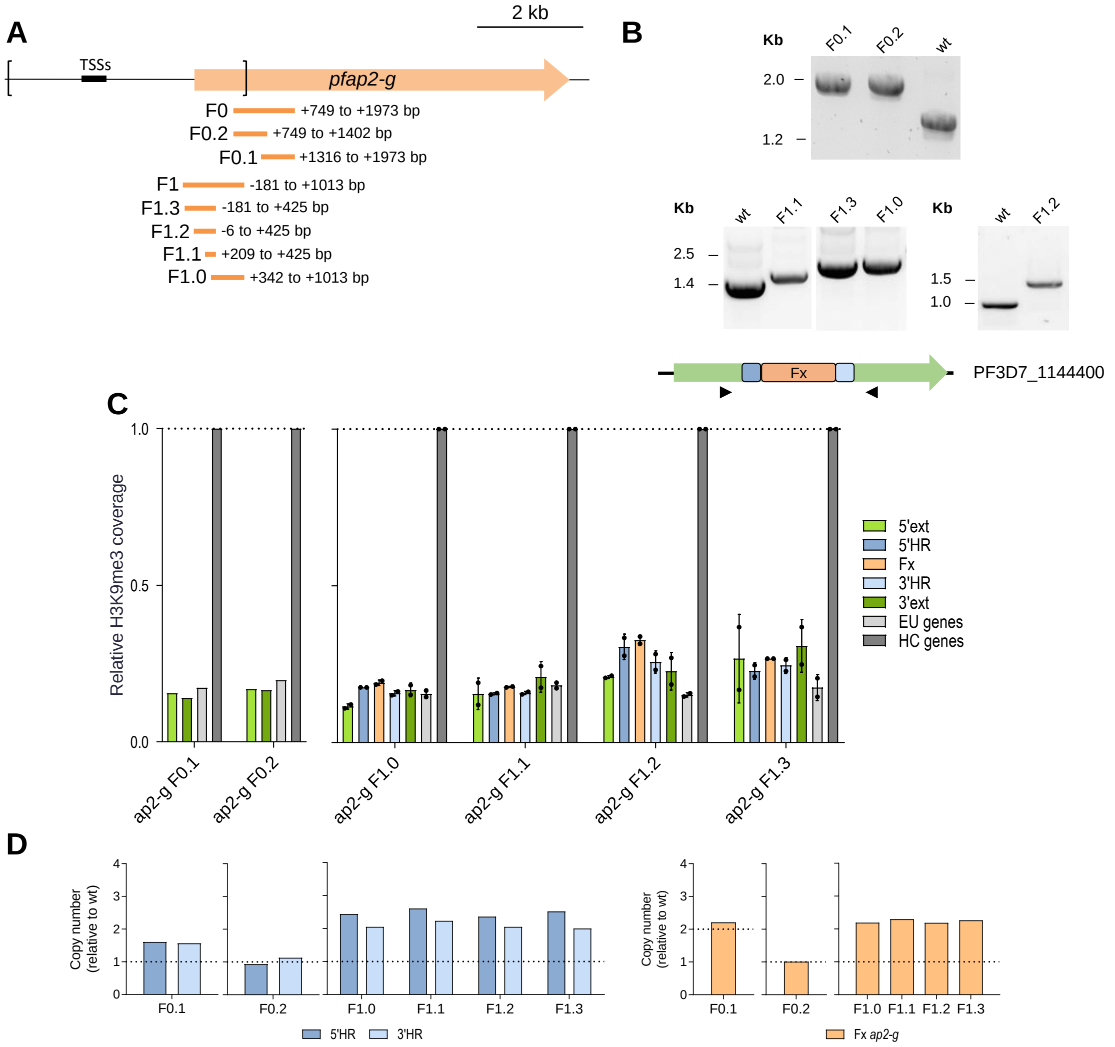

S3 Fig. Assessment of HC nucleation by small *pfap2-g* fragments derived from fragments F0 and F1

(A) Schematic (to scale) of the position of the small *pfap2-g* fragments derived from the F0 and F1 fragments.

(B) Diagnostic PCR confirming the correct integration of each fragment in the transgenic lines. The position of the PCR primers, external to the HRs, are shown in the scheme at the bottom.

(C) H3K9me3 ChIP-qPCR analysis of the transgenic lines carrying small (<1kb) *pfap2-g* fragments derived from *pfap2-g* F0 and F1 fragments. The position of the primers is as in main Fig 2A. Values are the % of the DNA recovered in the H3K9me3 IP (% input) at each position relative to the average % input in the positive control heterochromatic genes *var* PF3D7_1240300 and *clag3.2* ("HC genes"). "EU genes" are euchromatic genes *actI* and *serrs* used as negative controls (average of the two shown). The dashed line indicates the coverage in the positive control heterochromatic genes. Data are presented as the average and s.d. of two biological replicates (except for F0.1 and F0.2, N=1).

(D) qPCR analysis of the number of copies of the PF3D7_1144400 HRs (5'HR and 3'HR) and the fragments under analysis (Fx) in the gDNA of the transgenic

lines. Copy number was calculated relative to 1.2B (wild type) gDNA. The dashed lines indicate the expected value for single, correct integration.
