# Supplementary material for: Heterochromatin de novo formation and maintenance in Plasmodium falciparum
Source: PLoS Pathog. 2025 Jun 2;21(6):e1013137. doi: 10.1371/journal.ppat.1013137 (PMC12129197; doi:10.1371/journal.ppat.1013137)
Supplement: S4 Fig — (PDF) [file ppat.1013137.s004.pdf]

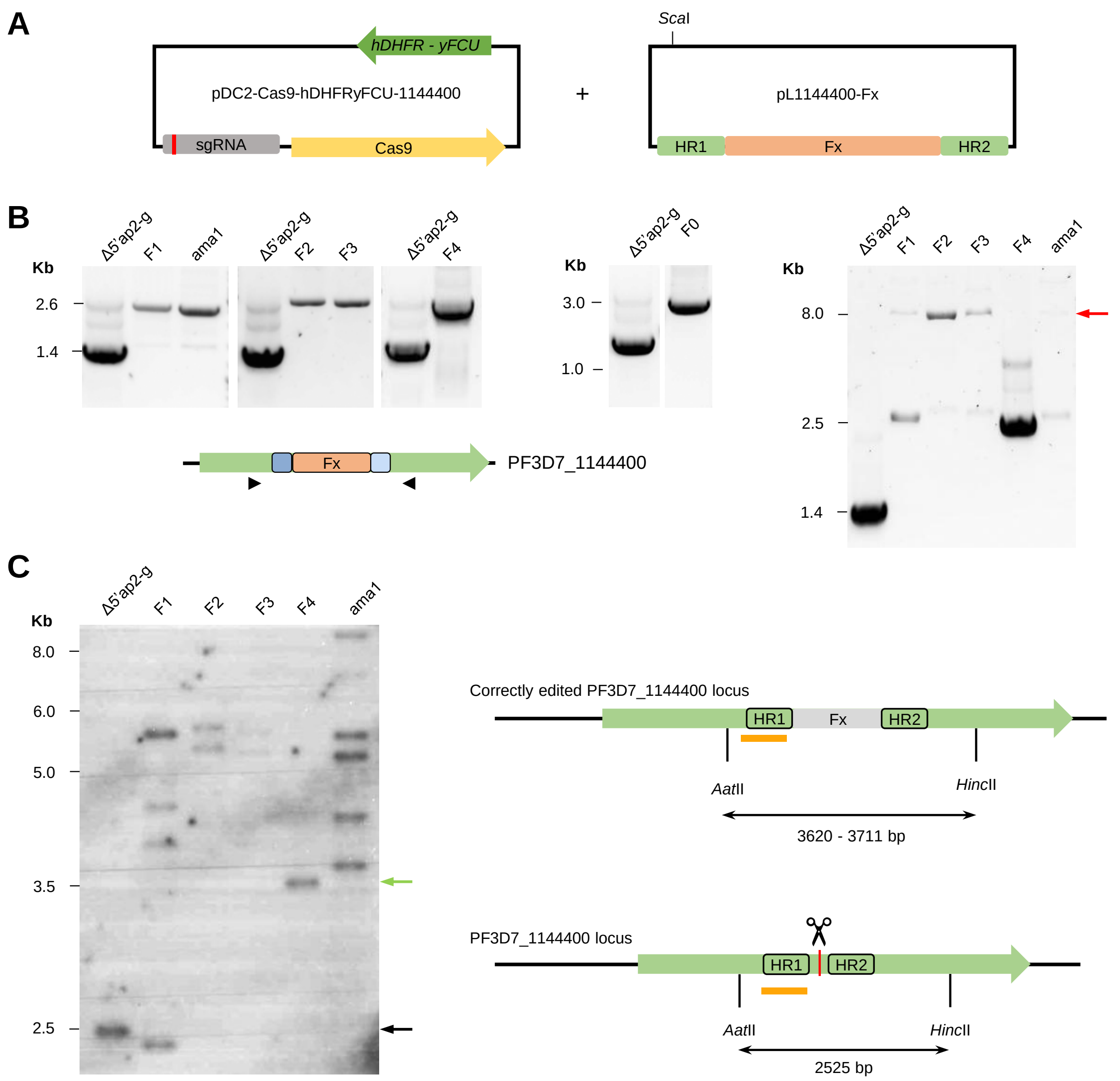

S4 Fig. Integration of ~1 kb fragments into the PF3D7_1144400 locus

(A) Schematic (not to scale) of the plasmids used to generate the transgenic lines carrying different *pfap2-g* ~1 kb fragments (F0 to F4) and an *ama1* fragment. Note that the *var* 1240300 transgenic line was generated with the plasmids described in S9 Fig (containing the *yfcu* negative selection marker) but for the experiments presented in main Fig 2 it was analyzed before negative selection, which makes it analogous to the other transgenic lines in these experiments.

(B) Diagnostic PCR confirming the correct integration of each fragment in the transgenic lines. The position of the PCR primers, external to the HRs, is shown in the scheme at the bottom. The panel at the right is a PCR amplification with the same primers using a longer extension time (10 min), which in most samples revealed a band that likely corresponds to integration of concatemers (red arrow).

(C) Southern blot analysis of the parental Δ5'ap2-g line (H11 subclone) and the transgenic lines with integrated fragments *pfap2-g* F1-F4 and ama1. The schematic shows the expected size of gDNA digested with *Aat*II, *Hinc*II and *Afl*II (the latter cleaving the plasmid but not the PF3D7_1144400 locus) and hybridized with a PF3D7_1144400-specific probe (orange line) in parasites with the fragments (Fx) correctly integrated (single copy) and in wild type parasites. The

scissors indicate the position targeted by the guide RNA, whereas black boxes indicate the position of the HRs. The green arrow indicates the approximate position of the bands expected for the correctly edited locus, and the black arrow the position of the band expected for the wild type locus. While the F4 line showed the expected band for correct single integration, other lines showed a complex pattern consistent with integration of different plasmid concatemer species.
