# Supplementary material for: Heterochromatin de novo formation and maintenance in Plasmodium falciparum
Source: PLoS Pathog. 2025 Jun 2;21(6):e1013137. doi: 10.1371/journal.ppat.1013137 (PMC12129197; doi:10.1371/journal.ppat.1013137)
Supplement: S5 Fig — (PDF) [file ppat.1013137.s005.pdf]

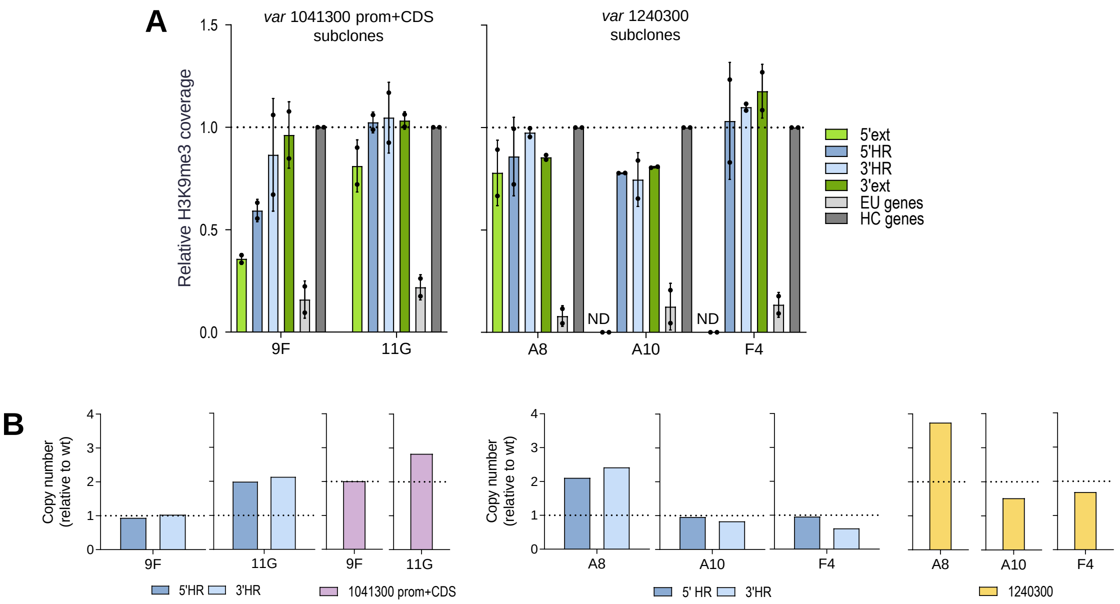

S5 Fig. Assessment of HC nucleation in subclones of parasite lines carrying *var* fragments

(A) H3K9me3 ChIP-qPCR analysis, as in S3C Fig, of subclones derived from two transgenic lines carrying *var* fragments (1041300 prom+CDS and 1240300). ND indicates "not detected", as some subclones carried a spontaneous deletion at that position. However, ectopic HC formation was observed in the subclone not carrying the deletion as well as in subclones carrying the deletion. Data are presented as the average and s.d. of two biological replicates.

(B) qPCR analysis of copy number, as in S3D Fig. The 1041300 prom+CDS subclones 9F and 11G carry single or multiple fragment integrations, respectively, but ectopic HC nucleation was observed in both subclones at similar levels.
