# Supplementary material for: Heterochromatin de novo formation and maintenance in Plasmodium falciparum
Source: PLoS Pathog. 2025 Jun 2;21(6):e1013137. doi: 10.1371/journal.ppat.1013137 (PMC12129197; doi:10.1371/journal.ppat.1013137)
Supplement: S6 Fig — (PDF) [file ppat.1013137.s006.pdf]

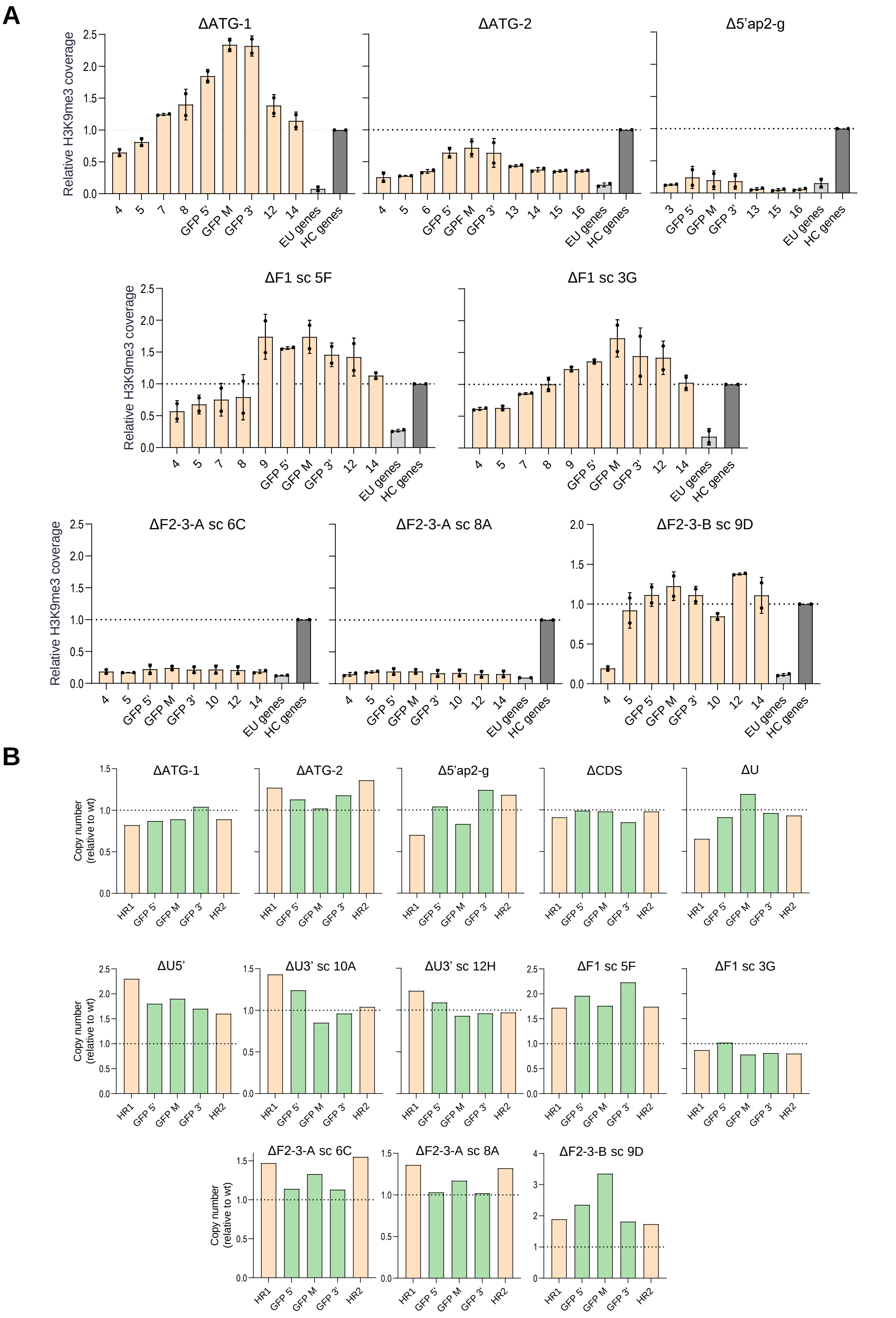

S6 Fig. Analysis of the role of different regions of *pfap2-g* in HC maintenance

(A) H3K9me3 ChIP-qPCR analysis of the ΔATG-1, ΔATG-2 and Δ5'ap2-g transgenic lines, and subclones (sc) from the ΔF1 and ΔF2-3 lines. Values are

the % input at each position relative to the % input in the positive control heterochromatic genes, as in S3C Fig. Numbers in the x axis are the primer pairs shown in main Fig 7A. The dashed line indicates the coverage in the positive control heterochromatic genes. Data are presented as the average and s.d. of two biological replicates.

(B) qPCR analysis of copy number, as in S3D Fig, of the transgenic lines in panel A and in main Fig 6. Copy number was determined for the different *pfap2-g* HRs used for each line and for three different regions of the inserted gfp*. The dashed lines indicate the expected value for correct, single integration.
