# Supplementary material for: Heterochromatin de novo formation and maintenance in Plasmodium falciparum
Source: PLoS Pathog. 2025 Jun 2;21(6):e1013137. doi: 10.1371/journal.ppat.1013137 (PMC12129197; doi:10.1371/journal.ppat.1013137)
Supplement: S7 Fig — (PDF) [file ppat.1013137.s007.pdf]

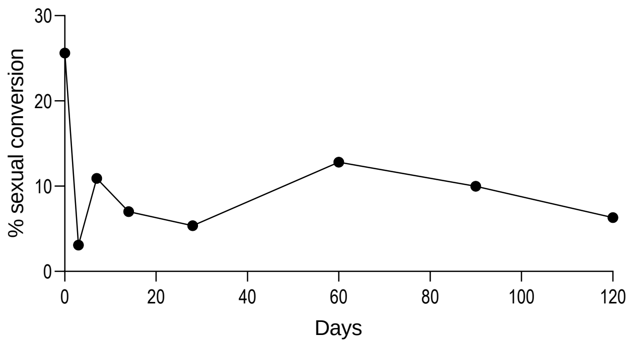

S7 Fig. Evolution of the sexual conversion rate of E5-AP2-G-DD after a second exposure to Shld1 at different times

Sexual conversion rate of the E5-AP2-G-DD line after induction with Shld1 for the first time (first data point, day 0), or after a second induction with Shld1 at different time points (cultures were maintained without Shld1 between the first and second exposures).
