# Supplementary material for: Heterochromatin de novo formation and maintenance in Plasmodium falciparum
Source: PLoS Pathog. 2025 Jun 2;21(6):e1013137. doi: 10.1371/journal.ppat.1013137 (PMC12129197; doi:10.1371/journal.ppat.1013137)
Supplement: S8 Fig — (PDF) [file ppat.1013137.s008.pdf]

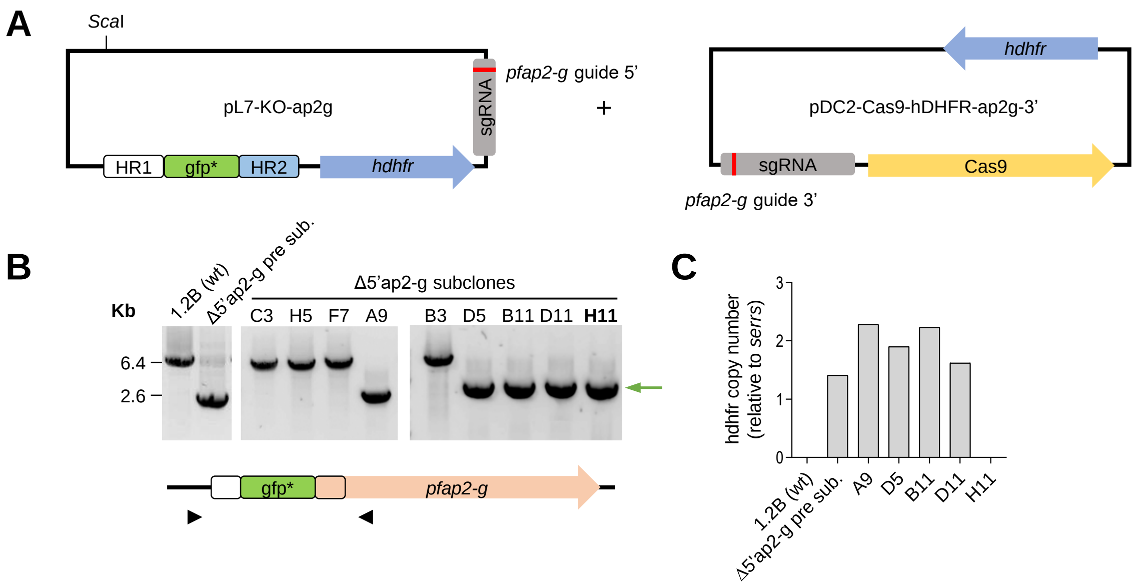

S8 Fig. Generation of the Δ5’ap2-g line

(A) Schematic (not to scale) of the plasmids used to generate the Δ5’ap2-g line.

(B) Diagnostic PCR analysis of the parental 1.2B line (1.2B wt), the Δ5’ap2-g line before subcloning and its subclones. The position of the band expected for correctly edited parasites is indicated by a green arrow. The schematic shows the position of the PCR primers, external to the HRs.

(C) qPCR analysis of copy number of the *hdhfr* selectable marker, relative to the *serrs* gene. Only the H11 clone carried the correct integration and was free of the marker. Therefore, this subclone was selected for all further experiments.
