# Supplementary material for: Heterochromatin de novo formation and maintenance in Plasmodium falciparum
Source: PLoS Pathog. 2025 Jun 2;21(6):e1013137. doi: 10.1371/journal.ppat.1013137 (PMC12129197; doi:10.1371/journal.ppat.1013137)
Supplement: S9 Fig — (PDF) [file ppat.1013137.s009.pdf]

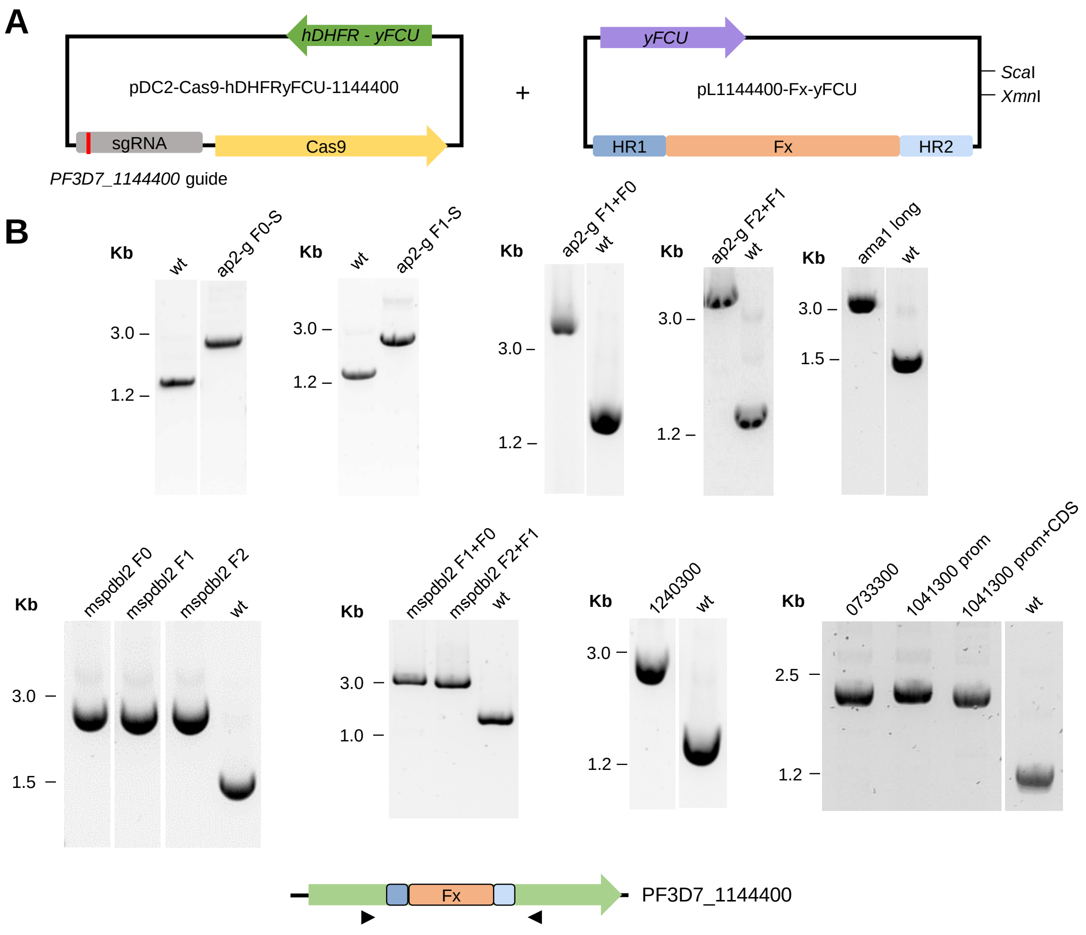

S9 Fig. Generation of transgenic lines to assess HC nucleation by single copies of integrated fragments

(A) Schematic (not to scale) of the plasmids used for the generation of transgenic lines with single copy fragment integrations, containing the *yFCU* negative selection marker.

(B) Diagnostic PCR of the transgenic lines generated using this strategy. The scheme at the bottom shows the position of the PCR primers, external to the HRs.
