# Supplementary material for: Heterochromatin de novo formation and maintenance in Plasmodium falciparum
Source: PLoS Pathog. 2025 Jun 2;21(6):e1013137. doi: 10.1371/journal.ppat.1013137 (PMC12129197; doi:10.1371/journal.ppat.1013137)
Supplement: S10 Fig — (PDF) [file ppat.1013137.s010.pdf]

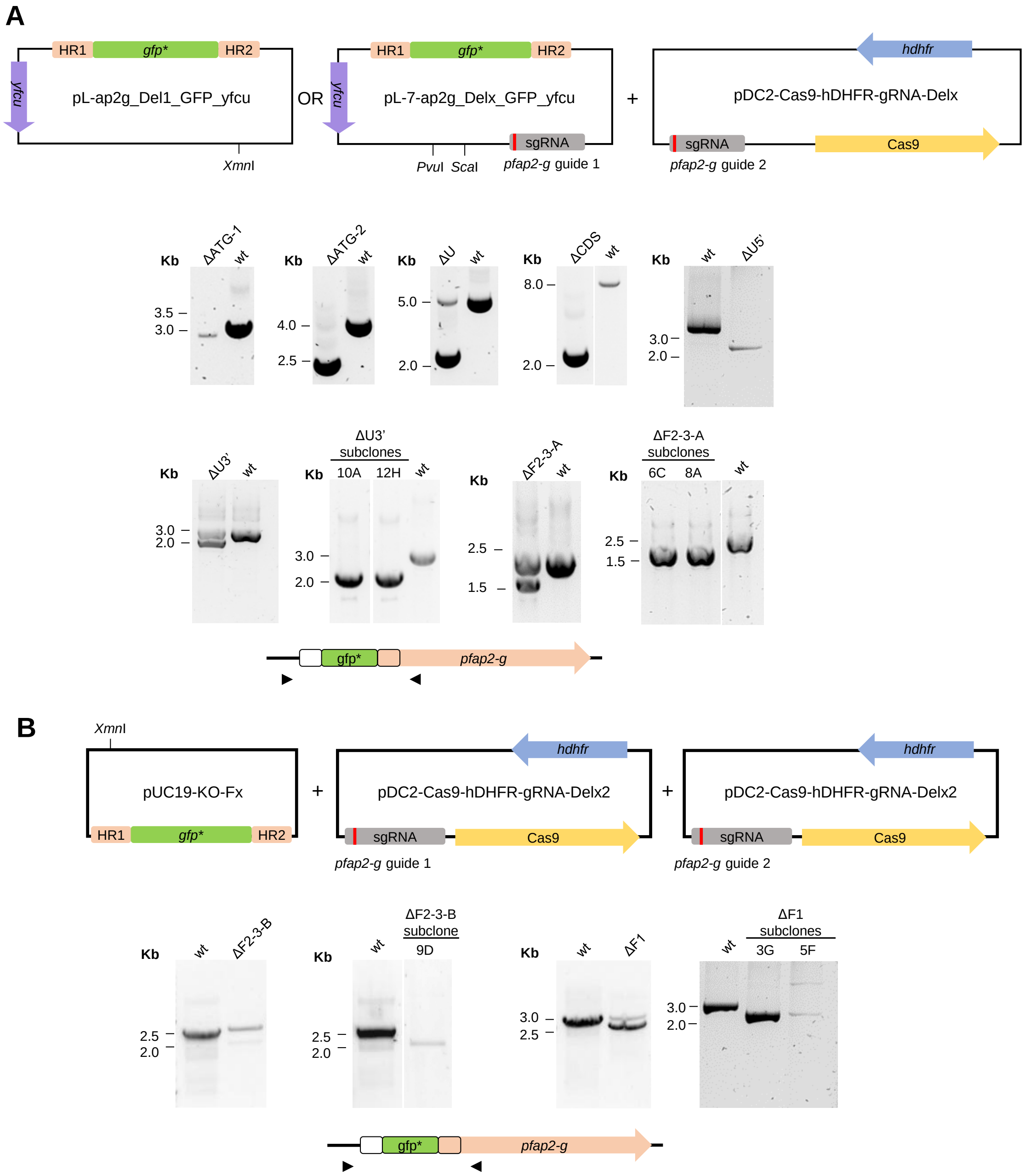

S10 Fig. Generation of the transgenic lines to study HC maintenance at the *pfap2-g* locus

(A) Top: Schematic (not to scale) of the plasmids used to delete different regions of the *pfap2-g* locus with a two plasmids strategy. The donor plasmid either lacked (ΔATG-1 line) or included (all other lines) a sgRNA expression cassette. Bottom: Diagnostic PCR to assess correct edition in the transgenic lines generated with this strategy. The scheme at the bottom shows the position of the PCR primers.

(B) Top: Schematic (not to scale) of the plasmids used to delete different regions of the *pfap2-g* locus using an alternative three plasmids strategy. Bottom: Diagnostic PCRs to assess correct edition in the transgenic lines generated with this strategy and their subclones (subclones were obtained to achieve pure populations of edited parasites).
