# Supplementary material for: Heterochromatin de novo formation and maintenance in Plasmodium falciparum
Source: PLoS Pathog. 2025 Jun 2;21(6):e1013137. doi: 10.1371/journal.ppat.1013137 (PMC12129197; doi:10.1371/journal.ppat.1013137)
Supplement: S1 Table — (PDF) [file ppat.1013137.s011.pdf]

S1 Table. Oligonucleotides used in this study. Sequence of the oligonucleotides used in this study, classified according to their use.

Oligonucelotides used for 5'RACE	
**Name**	**Sequence (5'-3')**
5'RACE_RNA_adapter	GCUGAUGGCGAUGAAUGAACACUGCGUUUGCUGGC
5'RACE_Outer	GATGGCGATGAATGAACACTG
Pfap2-g_+345_R	GATACATTCTCGTTACTCTGCA
5'RACE_Inner	GAATGAACACTGCGTTTGCTG
Pfap2-g_+272_R	CATGCTCTCTTCCCATTCGAA

Primers for cloning steps	
**Name**	**Sequence (5'-3')**
Pfap2g_-3929_HR1_KO_F_NotI	tggtgtgcggccgcCGTACATATATACAAATAGAGAT
Pfap2g_-3521_HR1_KO_R_PstI	tggtgtctgcagACAGGTATTGTACGCCTTTTA
Pfap2g_+991_HR2_KO_F_SpeI	tgttggactagtCCTTGAAAAGAATATAGAAGAAC
Pfap2g_+1973_HR2_KO_R_AflII	tggtgtcttaagCAATTTATCAGCATCGTCATCA
GFP_+3_F_PstI	tggtgtctgcagAGTAAAGGAGAAGAACTTTTCA
GFP_+714_R_SpeI	tggtgtactagtTTATTTGTATAGTTCATCCATGC
Pfap2g_-3503_Guide_F	taagtatataatattTTCTTTTTAAGGTTGCGTACgttttagagctagaa
Pfap2g_-3522_Guide_R	ttctagctctaaaacGTACGCAACCTTAAAAAGAAatattatatactta
Pfap2-g_+940_Guide_F	taagtatataatattTCATAAGAAATATGATTCATgttttagagctagaa
Pfap2-g_+921_Guide_R	ttctagctctaaaacATGAATCATATTTCTTATGAaatattatatactta
1144400_+1144_HR1_F_SacII	tggccgcggGGAAAGACAATCAACAAACCTT
1144400_+1562_HR1_R_SpeINcoI	tggtgtactagtctgccatggTCACTTGAAATCGTTTTGTTATTA
1144400_+1646_HR2_F_SpeI	tggttgactagtATTGTATAAATGAAGAGACATACA
1144400_+2199_HR2_R_AflII	tggtgtcttaagTGATTATTATGTGTAACTTCAGTA
ap2g_-181_F1_F_NcoI	tggtgtccatggGTAGGTACATTCAAATATCTCC
ap2g_+992_F1_R_SpeI	tggtgtactagtTTCTTCTATATTCTTTTCAAGGAT
ap2g_-1200_F2_F_NcoI	tggtgtccatggGATACTTTATGATTAGTTGATATG
ap2g_+118_F2_R_SpeI	tggagaactagtTTCCTGGGATGTAATCAAAAGT
ap2g_-2147_F3_F_NcoI	tggtgtccatggATAATGCTCTGAGAATATATACTT
ap2g_-891_F3_R_SpeI	tgttggactagtAATTTATATATCTCGACACCTTTA
ap2g_-3000_F4_F_NcoI	tggtgtccatggTATTGACGTGCATACTATATGTA
ap2g_-1834_F4_R_SpeI	tggagaactagtAATGTATTTCATGATATTAGTGTAA
ama1_-966_F_NcoI	tggtgtccatggGTTATGTGTAGAATATTTACAAAG
ama1_+249_R_SpeI	tgttggactagtACCTTCGTGGTCTATTGGATA
Pfap2-g_+425_nucleo_R_SpeI	tggtgtactagtTCTATGATTTAACAACATATCCAA
Pfap2-g_-6_2nucleo_F_NcoI	tggtgtccatggAAGAAGATGACCGCTAAGATAT
Pfap2-g_+210_1nucleo_F_NcoI	tggtgtccatggATTGAAGAAAGTTGTTTTGACATT
Pfap2-g_+341_RestF1_F_NcoI	tggtgtccatggTTGCAGAGTAACGAGAATGTAT
Pfap2-g_+748_F0_F_NcoI	tggtgtccatggGAAATGTAAAAGAGTATTCTCATA
Pfap2-g_+1951_F0_R_SpeI	tggtgtactagtCAATTTATCAGCATCGTCATCA
1144400_+1566_Guide_F	taagtatataatattATTATGTGTTCCAAGAACGAgttttagagctagaa
1144400_+1585_Guide_R	ttctagctctaaaacTCGTTCTTGGAACACATAATaatattatatactta
PFL1950W_-1096_F_NcoI	tggtgtccatggGACATTAGTTATATGAATATATTGG
PFL1950W_+112_R_SpeI	acaccaactagtGCATCTCTTTGTACTTCTGCG
AMA1_-1692_F_NcoI	tggtgtccatggTATACGAGTAAAACACATCACAG
AMA1_+250_R_SpeI	acaccaactagtGTTCTTGTGGTGCGGGTTCG
mspdbl2_+705_NcoI_F	tggtgtccatggTCATATGCATCATCTGAAGC
mspdbl2_+1883_SpeI_R	acaccaactagtTCTATTTCCCCATCTGTATTA
mspdbl2_-173_NcoI_F	tggtgtccatggTATTTGTTCATTGAACTGTTAATG
mspdbl2_+1004_SpeI_R	acaccaactagtGTACATTGGTTATCTTTCTGC
mspdbl2_-1013_NcoI_F	tggtgtccatggGATAAGTCGCTGCGATTCC
mspdbl2_+120_SpeI_R	acaccaactagtTATGTTATTTCTTAAATTAGGGT
varB_-1412_NcoI_F	tggtgtccatggCAAGGTAATTTCATACATATGTG
0733000_-1_speI_R	acaccaactagtTTTCGTTATTGGTGCACTACAT
1041300_-1_speI_R	acaccaactagtTGTACCGACAACATGATGTTAT
1041300_-1096_F_NcoI	tggtgtccatggTGATTGAAGAATACGTATGCCT
1041300_+148_R_SpeI	acaccaactagtACAAACGTCCATGCAATTGAC
Pfap2-g_-448_HR1_KOF1_F	cggtacccggggatccATATGTCCTATAGGTGTCAAAC
Pfap2-g_-159_HR1_KOF1_R	tcttctcctttactctgcagGGAGATATTTGAATGTACCTAC
Pfap2-g_+993_HR2_KOF1_F	atacaaataaactagtCCTTGAAAAGAATATAGAAGAAC
Pfap2-g_+1974_HR2_KOF1_R	gactctagaggatccCAATTTATCAGCATCGTCATCA
Pfap2-g_-159_Guide_F	ttctagctctaaaacTTATATTGGCACTAATTTAGaatattatatactta
Pfap2-g_-139_Guide_R	taagtatataatattCTAAATTAGTGCCAATATAAgttttagagctagaa

Primers for cloning steps	
**Name**	**Sequence (5'-3')**
Pfap2-g_-1857_HR1_KOF2-3_F	cggtacccggggatccTTACACTAATATCATGAAATACATT
Pfap2-g_-1427_HR1_KOF2-3_R	tcttctcctttactctgcagACAAAATCTATAATCTTATATATGAA
Pfap2-g_-129_HR2_KOF2-3_F	atacaaataaactagtAATTTGAAGTACCAACATATACAT
Pfap2-g_+234_HR2_KOF2-3_R	gactctagaggatccAATGTCAAAACAACTTTCTTCAAT
Pfap2-g_-1412_Guide_F	taagtatataatattTTTAATAATACGTATGCTTGgttttagagctagaa
Pfap2-g_-1393_Guide_R	ttctagctctaaaacCAAGCATACGTATTATTAAAaatattatatactta
ap2g_-479_InF_SacII_F	actaaatatatatccaatggccgcggCTTTAATGTTGTATGTATGTTTA
ap2g_-1_InF_SpeI_HindIII_R	actagtacacaccaaagcttCTTCTTAAATATTCCTTATTAAAA
ap2g_+993_InF_HindIII_SpeI_F	aagctttggtgtgtactagtCCTTGAAAAGAATATAGAAGAAC
ap2g_+1974_InF_EcoRI_R	ttccccgaaaagtgccacctgaattcCAATTTATCAGCATCGTCATCA
ap2g_-1402_InF_SacII_F	actaaatatatatccaatggccgcggCGTATGCTTGTGGATATTATAA
ap2g_-900_InF_HindIII_AflII_R	aagcttacacaccacttaagTCTCGACACCTTTATTATATATA
ap2g_+1493_InF_AflII_HindIII_F	cttaagtggtgtgtaagcttATAAGAAGAACAATAAAAATAAGA
ap2g_+1981_InF_NcoI_R	gtgttattatttttaccgttccatggTATTATCCAATTTATCAGCATC
ap2g_-3929_InF_SacII_F	actaaatatatatccaatggccgcggCGTACATATATACAAATAGAGAT
ap2g_-3522_InF_HindIII_AflII_R	aagcttacacaccacttaagACAGGTATTGTACGCCTTTTA
ap2g_+1_InF_AflII_HindIII_F	cttaagtggtgtgtaagcttATGACCGCTAAGATATTAAATC
ap2g_+521_InF_NcoI_R	gtgttattatttttaccgttccatggATATACGCTCATTTCCTTTATC
ap2g_+493_InF_SacII_F	actaaatatatatccaatggccgcggAAAGAGGATAAAGGAAATGAG
ap2g_+984_InF_HindIII_AflII_R	aagcttacacaccacttaagCATATTACCTATATAAGTTTTAG
ap2g_+7299_InF_AflII_HindIII_F	cttaagtggtgtgtaagcttATATCATCCTTTTTTTTTATAAAAT
ap2g_+7744_InF_NcoI_R	gtgttattatttttaccgttccatggACCAAGTTCATTTTAATGTTATT
GFP_+3_AflII_F	tggtgtcttaagAGTAAAGGAGAAGAACTTTTC
GFP_+714_HindIII_R	acaccaaagcttTTATTTGTATAGTTCATCCATG
GFP_+3_HindIII_F	tggtgtaagcttAGTAAAGGAGAAGAACTTTTC
GFP_+714_SpeI_R	acaccaactagtTTATTTGTATAGTTCATCCATG
ap2g_+45_guide1_F	taagtatataatattAGGGTATACAGGGATATCGAgttttagagctagaa
ap2g_+45_guide1_R	ttctagctctaaaacTCGATATCCCTGTATACCCTaatattatatactta
ap2g_-827_guide1_F	taagtatataatattTATGATATCAAATTAAAATAgttttagagctagaa
ap2g_-827_guide1_R	ttctagctctaaaacTATTTTAATTTGATATCATAaatattatatactta
ap2g_+1453_guide2_F	taagtatataatattAAAGATGAACACAAGAAGGAgttttagagctagaa
ap2g_+1453_guide2_R	ttctagctctaaaacTCCTTCTTGTGTTCATCTTTaatattatatactta
ap2g_-199_guide2_F	taagtatataatattGTAACTATCTATATATAAGTgttttagagctagaa
ap2g_-199_guide2_R	ttctagctctaaaacACTTATATATAGATAGTTACaatattatatactta
ap2g_+1104_guide1_F	taagtatataatattTTATAGTAATCAAACATGCAgttttagagctagaa
ap2g_+1104_guide1_R	ttctagctctaaaacTGCATGTTTGATTACTATAAaatattatatactta
ap2g_-1427_InF_HindIII_AflII_R	aagcttacacaccacttaagACAAAATCTATAATCTTATATATGAA
ap2g_-130_InF_AflII_HindIII_F	cttaagtggtgtgtaagcttAATTTGAAGTACCAACATATACAT
ap2g_+234_InF_NcoI_R	gtgttattatttttaccgttccatggAATGTCAAAACAACTTTCTTCAAT
ap2g_-1633_gRNA_R	taagtatataatattAAAAGTATTTATATCTCTCAgttttagagctagaa
ap2g_-1633_gRNA_F	ttctagctctaaaacTGAGAGATATAAATACTTTTaatattatatactta
ap2g_-1402_InF_AflII_HindIII_F	cttaagtggtgtgtaagcttCGTATGCTTGTGGATATTATAA
ap2g_-900_InF_NcoI_R	gtgttattatttttaccgttccatggTCTCGACACCTTTATTATATATA

Primers for diagnostic PCR	
**Name**	**Sequence (5'-3')**
1144400_+865_F	GATATTATGAGTACTGATAGTGA
1144400_+2319_R	TGACTCGTTCAGGTTATTCTTA
1144400_+1799_R	CAGATCTATATTTCATTTGTTCTT
Pfap2-g_-2147_UpTSS1_F	TAATGCTCTGAGAATATATACTTA
Pfap2-g_UpTSSR_-710	TGTATGTAAAAATAAAGCACCAC
Pfap2-g_CDS_+345_R	GATACATTCTCGTTACTCTGC
Pfap2-g_5'UTR_-460_F	GCTTCTTTAATGTTGTATGTATG
Pfap2-g _+2474_R	TTACTCCATTAGGTGCATTCAT
1144300_+4_F	CACATGGAGCAAGCAGGTAT
1144300_+632_R	TTTTGTAAACGTCTGGTTCTCT
1144300_UTR_F	TAACAAATATGAATATTGAAGGATA
1144400_-904_F	TAAGTATGGTTCATTTAGGGGT
1144400_+22_R	TGTCGTCTTTAATCAGTTCCAT
Pfap2-g_UpTSSF_ -1820	ATGGCTTTTATTTATTCTTAATTGT
Ap2g_3' EXT KO1KO2_R_+2277	CAAACGGTTACTATTATTACTATT
Ap2g_5' EXT KO3_F_-4300	GAAAATTAAACATTGGAGAATTGA
Ap2g_3' EXT KO3_R_+755	CATTTCATCAAAGACGCCGTA
Ap2g_5' EXT KO4_F_+200	GTATGAAATAATTGAAGAAAGTTG
Ap2g_3' EXT KO4_R_+8133	GTACAACAAAAACTGAACTCTTT

**Primers for gDNA qPCR and ChIP-qPCR**	
**Name**	**Sequence (5'-3')**
Pfap2-g_-2147_UpTSS1_F	TAATGCTCTGAGAATATATACTTA
Pfap2-g_-1979_UpTSS1_R	CAATTAGGATAAATAAATATTCCAT
Pfap2-g_ -1820_UpTSS2_F	ATGGCTTTTATTTATTCTTAATTGT
Pfap2-g_ -1644_UpTSS2_R	TATCTCTCACGGTTCATATCTT
Pfap2-g_-1409_DwnTSS_F	TAATACGTATGCTTGTGGATATT
Pfap2-g_-1226_DwnTSS_R	ATATGGAAACTAATAATAAATTGTTA
PF3D7_1222600_F1 (CDS)	AACAACGTTCATTCATTCAATAAATAAGG
PF3D7_1222600_R1 (CDS)	ATGTTAATGTTCCCAAACAACCG
Seryl_qPCR_F (serrs)	AAGTAGCAGGTCATCGTGGTT
Seryl_qPCR_R (serrs)	TTCGGCACATTCTTCCATAA
hdhfr_F	AGTAGAAGGTAAACAGAATCT
hdhfr_R	GGCATCATCTAGACTTCTGG
plasmid_3_F	ATGTATCCGCTCATGAGACAA
plasmid_3_R	TGAGCAAAAACAGGAAGGCAA
5'HR_1144400_+1424_F	TAATGCAAAACGATAGTAATCTTA
5'HR_1144400_+1526_R	TCTTCATTAGGATCATATCCATT
3'HR_1144400_+1676_F	TGAAACTTGAAAACTGTGATGAA
3'HR_1144400_+1799_R	CAGATCTATATTTCATTTGTTCTT
PFL1950wF	CTATGTTGTATTATTCGATATTTTC
PFL1950wR	AGAATAGGAAAATACAAATTATAGC
Pfap2-g_CDS_+259F	GAAGAGAGCATGCAATGAAGT
Pfap2-g_CDS_+382R	TTGTCCATGCAACTATTCGATA
Pfap2-g_-1409_DwnTSS_F	TAATACGTATGCTTGTGGATATT
Pfap2-g_-1226_DwnTSS_R	ATATGGAAACTAATAATAAATTGTTA
Ap2-g_-2967_F	ATTATTACCTTCGGTACCTTAAT
Ap2-g_-2861_R	AATGCACTTTTTGAGTACAGTTA
PFL1085w_-2553_A2.5F	CAATATAATACCATACTTCAAAAC
PFL1085w_-2460_A2.5R	AGTATGGTTTTGTACTCCTTTTA
AMA1_+23_F	ATTATTGAGCGCCTTTGAGTTT
AMA1_+145_R	GTGTAATGGATATTCGTATTCTT
Pfap2-g_+653_F	TTTCCCATACTATATGCTGAAAT
Pfap2-g_+774_R	CATATGAGAATACTCTTTTACATT
Pfap2-g_+754_F	GTAAAAGAGTATTCTCATATGTAA
Pfap2-g_+876_R	AACATAAAAGTACATGACATCATT
clag3.2_5_F	TAGGCGAAAATAAAAACGAAAATG
clag3.1_clag3.2_5_R	CATGGATTTTAATTGTTCAATATTG
5'ext_1144400_+734_F	ATGAAGTAGCTACTACAATACAT
5'ext_1144400_+870_R	AATATCCATAAGAACATCCTTGT
3'ext_1144400_+2177_F	CTGAAGTTACACATAATAATCAAA
3'ext_1144400_+2319_R	TGACTCGTTCAGGTTATTCTTA
mspdbl2_-697_F	TCATGTATATGTAAAAGTGATTCT
mspdbl2_-482_R	CCCTCAAATAAAGTGCATTATG
mspdbl2_+766_F	CTCATCAAGCTATAAGATATAGT
mspdbl2_+966_R	CATCATTGCTTCCCAAACATG
Pfap2-g_-2147_UpTSS1_F	TAATGCTCTGAGAATATATACTTA
Pfap2-g_-1979_UpTSS1_R	CAATTAGGATAAATAAATATTCCAT
PF3D7_1222600_F1	AACAACGTTCATTCATTCAATAAATAAGG
PF3D7_1222600_R1	ATGTTAATGTTCCCAAACAACCG
1041300_-129_F	TCTACCATATTACAATACTCCC
1041300_-9_R	ACATGATGTTATACAATTTGGTG
0733000_-1013_F	TGGTATCAAGAATACGTATGCT
0733000_-842_R	GAACAAGAACGTATCGATATAG
Ap2g_3' EXT KO1KO2_F _+2088	CAACCTAACGTTCTTGAAAAAG
Ap2g_3' EXT KO2KO2_R_+2277	CAAACGGTTACTATTATTACTATT
Ap2g_5' EXT KO3_F_-4300	GAAAATTAAACATTGGAGAATTGA
Ap2g_5' EXT KO3_R_-4138	TTACATATACATATACATACATTTA
Ap2g_HR1 KO3_F_-3902	GCTAGACGTACAATAATATACAA
Ap2g_HR1 KO3_R_-3552	TTTTTTATTATTTGCTATTTTTAAAG
ap2g_-805_R	GAAACCTTTTGAATTTATAATATTAT
ap2g_-1547_F	GATAAAGTAGAATATTGTTCTATAT
ap2g_-1392_R	TGCATATGTATAAATATTGTCAGT
ap2g_-3760_F	GCACATATATTTTAATTAGCTAGA
ap2g_-3708_F	CTTTATATATTGTATTTATGCATAT
ap2g_-3757_R	GTGCACAAGATTAATAATAATTAT
ap2g_-3523_R	ACAGGTATTGTACGCCTTTTAA

Primers for gDNA qPCR and ChIP-qPCR	
**Name**	**Sequence (5'-3')**
ap2g_-5004_F	CTGAATATTTTTCTTTTGGTCGT
ap2g_-4861_R	GACATAATATATATTGAGCATGC
ap2g_-4359_F	AATAGATAAAGTAGAATATTGTTCT
ap2g_-4200_R	GCATATGTATAAATATTGTCAGTT
ap2g_-3930_F	CGTACATATATACAAATAGAGATT
ap2g_-3737_R	TCTAGCTAATTAAAATATATGTGC
Pfap2-g_UpTSSF_ -1820	ATGGCTTTTATTTATTCTTAATTGT
ap2g_-1321_F	GAATGTTGTAGTTCTTTCTTTCT
ap2g_-1177_R	CATATCAACTAATCATAAAGTATC
Pfap2-g_qPCR_-563_R	TGCAAAAACATGCACATAATATG
Pfap2-g_qPCR_-484_F	GCTTCTTTAATGTTGTATGTATG
Pfap2-g_qPCR_-327_R	GTTAGTAATACAAAAATGTAAATAC
ap2g_-352_F	GTATTTACATTTTTGTATTACTAAC
ap2g_-160_R	GGAGATATTTGAATGTACCTAC
Ap2g_5' EXT KO4_F_+200	GTATGAAATAATTGAAGAAAGTTG
Pfap2-g_CDS_+345_R	GATACATTCTCGTTACTCTGC
Ap2g_3' EXT KO3_F_+618	CATCTATTTATTGTCAAGAAAGAA
Ap2g_3' EXT KO3_R_+755	CATTTCATCAAAGACGCCGTA
PfAP2-G +1106 F	TAGTAATCAAACATGCATGGATT
PfAP2-G +1237 R	CAACTACAGGTACAGAACATAT
Ap2g_HR2 KO2_F_+1721	TTCTCATTCTTCAAATAGCCTTG
Ap2g_HR2 KO2_R_+1866	TTTAACATTATCTTTGTGAGAATAA
ap2g_+2017_IPC_F	GATTATTTTACTATTTGTGATCCT
ap2g_+2183_IPC_R	TGATTACTTATAGAATTATGCTTG
pfap2-g_+3979_R	ATGTTAATGTTCCCAAACAACCG
pfap2-g_+3874_F	AACAACGTTCATTCAATAAATAAGG
PFL1085w_F2	GCTACCTTACTAATGTATTAAGTC
PFL1085w_R2	CATATTATTAAAGAATTGGTTCACG
PFL1085w_+7357_F3	CCTTTTGAATATTCCCTATAACT
PFL1085w_+7467_R3	GTTATTTAAAAATGTCACAAGATG
Ap2g_3' EXT KO4_F_+7981	TTTTCCACATTATAAGAACTGTAT
Ap2g_3' EXT KO4_R_+8133	GTACAACAAAAACTGAACTCTTT
ap2g_+26_F	TCGATATCCCTGTATACCCTT
ap2g_+224_R	CAACTTTCTTCAATTATTTCATAC
GFP_+1_F	GTAAAGGAGAAGAACTTTTCAC
GFP_+158_R	GGTAGTTTTCCAGTAGTGCAA
GFP_+580_F	TTACCAGACAACCATTACCTG
GFP_+714_R	TTATTTGTATAGTTCATCCATGC
GFP_+348 _F	GTGATACCCTTGTTAATAGAAT
GFP_+540_R	ATGGTCTGCTAGTTGAACGC
