# Supplementary material for: Heterochromatin de novo formation and maintenance in Plasmodium falciparum
Source: PLoS Pathog. 2025 Jun 2;21(6):e1013137. doi: 10.1371/journal.ppat.1013137 (PMC12129197; doi:10.1371/journal.ppat.1013137)
Supplement: Attachment — Submitted filename: PerezCantero_etal_Rebuttal.pdf [file ppat.1013137.s014.pdf]

PLEASE NOTE: LINE NUMBERS ARE FOR THE TRACK CHANGES VERSION

Part I – Summary

Please use this section to discuss strengths/weaknesses of study, novelty/significance, general execution and scholarship.

Reviewer #1: In this manuscript, the authors investigate DNA sequence determinants of heterochromatin nucleation/maintenance in P. falciparum, which has been largely unexplored. They provide novel and well-controlled evidence that specific sequences from var genes and ap2-g are able to nucleate heterochromatin at a normally euchromatic locus. They go on to elucidate which sequences are needed at the endogenous ap2-g locus to maintain heterochromatin. It is interesting how the data support different roles – HC nucleation versus maintenance – for different DNA elements. I appreciate the difficulty of all these experiments, especially the genome editing in AT-rich regions, incorporation of multiple copies, etc. The authors were very careful in their experiments, always aware of the limitations of their system, and never overstate their findings. The authors readily admit the main weakness I found with this study, being that the fragments investigated were too long to provide insight into which DNA-binding factors or ncRNAs might facilitate their described heterochromatin nucleation/maintenance phenotypes. However, the data are convincing/conclusive, interesting, and provide solid evidence for the role of these DNA elements in heterochromatin formation in the most "real" way possible (i.e. direct modification of endogenous loci, without the use of artificial chromosomes, etc). I feel that their findings would be useful to the community studying epigenetics at large, and particularly for those interested in heterochromatin regulation in P. falciparum.

We thank the Reviewer for appreciating the importance and interest of our results, and also the difficulties of this work and our efforts to avoid overstating the findings.

Reviewer #2: The authors have undertaken a thorough study investigating a difficult question that has long puzzled malaria researchers. Their demonstration of sequence dependent nucleation and maintenance of heterochromatin is important and useful.

We thank the Reviewer for appreciating the thoroughness and importance of our experiments and results.

Reviewer #3: In this study, the authors aimed to understand how heterochromatin is nucleated and maintained in the human malaria parasite P. falciparum. Heterochromatin in malaria parasites is critical for controlling key pathogenicity mechanisms such as antigenic variation and several erythrocyte invasion ligands. Another key mechanism directed by heterochromatin is the developmental decision between asexual and sexual parasite fate, which is controlled by the transcription factor AP2-G that is heterochromatin packaged in asexual parasites, but upon destabilization of heterochromatin at its promoter becomes activated and triggers the sexual differentiation program. To understand the sequence requirements for heterochromatin nucleation with a focus on AP2-G, the authors integrated various sequence elements form the AP2-G coding and promoter region (also some other control genes) into a non-essential euchromatic gene locus and assayed the heterochromatin state by chromatin immunoprecipitation. In addition, to gain insight into heterochromatin maintenance, they also modified the endogenous AP2-G locus to define sequences required for maintenance of the pre-established heterochromatin status. This led to the narrowing down of two relatively broad and overlapping regions that are supporting nucleation and maintenance, respectively. However, the authors also demonstrate that

there is significant clonal variability, indicating that these sequences are not strictly driving these processes.

We thank the Reviewer for the precise summary of our main findings.

Part II – Major Issues: Key Experiments Required for Acceptance
Please use this section to detail the key new experiments or modifications of existing experiments that should be absolutely required to validate study conclusions.

Generally, there should be no more than 3 such required experiments or major modifications for a "Major Revision" recommendation. If more than 3 experiments are necessary to validate the study conclusions, then you are encouraged to recommend "Reject".

Reviewer #1: I appreciate that the authors discuss probabilistic heterochromatinization of loci such as ap2-g. The authors see quite a bit of variability between clones in this study. In Fig. 6E, two different clones of delta U3' show very different phenotypes. This is true for the sexual conversion experiments as well. This suggests that H3K9me3 levels are rather dynamic, and that maybe at certain loci, it's not simply a question of euchromatin versus heterochromatin, but dynamism of H3K9me3 levels…more like a constant balance between the writers and erasers. However, I think the authors down-play their findings a bit with regard to heterochromatin nucleation driven by ap2-g upstream sequences. Yes, the var gene sequences are more efficient at nucleating heterochromatin, but I would not draw the conclusion that "nucleation at the pfap2-g locus [is not] a deterministic event in which the presence of specific sequences always results in formation of HC". In the AP2-G-DD background, I'm assuming that the entire upstream region of ap2-g is present, including the sequence that the authors determined was sufficient to drive heterochromatin nucleation at the 1144400 gene. Although lower than the H3K9me3 levels at ap2-g in D7, B11 still has plenty of H3K9me3 there and does not show 100% conversion. Thus, I would not say that this locus is euchromatinized in B11. The authors claim in the discussion that heterochromatin re-establishment at ap2-g in this line takes multiple generations, but they do not provide H3K9me3 ChIP time course data to support this. The key experiment to support the claims that ap2-g cis sequences are not deterministic would be to delete the upstream HC-nucleating sequence of ap2-g in clone B11 to see if this would truly deplete H3K9me3 levels at the locus and lead to 100% conversion.

We agree with the Reviewer that there was variability between subclones and this reflects the dynamic nature of heterochromatin as a balance between opposing enzymatic activities. Indeed, our data and previously published studies show that some chromosomal sequences are not always found as heterochromatin or always found as euchromatin, supporting the view that this is a non-deterministic event. However, some transfected lines showed intermediate heterochromatin levels before subcloning, but subclones (reflecting the patterns in individual parasites, largely conserved in their progeny) showed rather fully heterochromatic or fully euchromatic patterns (*e.g.*, Fig. 4A,C and 6E and ref. 16, PMID 36515553), indicating that intermediate levels largely reflect heterogeneity between individual parasites in the bulk population.

The Reviewer is right in the appreciation that even if heterochromatin nucleation by *pfap2-g* sequences is less efficient than by *var* sequences, heterochromatin ends up forming when the full *pfap2-g* sequence is present, even though this requires multiple generations and does not occur in all individual parasites. To make this clear, we

modified the statement mentioned by the Reviewer in the Discussion (**changes in lines 507-13**) to make clear that non-deterministic nucleation refers to specific fragments and that heterochromatin ends up forming when the full locus is present.

The entire *pfap2-g* upstream region is indeed present in the AP2-G-DD line, as endogenous C-terminal tagging was achieved by plasmid integration at the end of the gene (Sup. Fig. 6 from PMID 24572369,). This is now explained in the text (**changes in line 414**). We also agree with the Reviewer that we do not provide direct evidence for the euchromatic state of the *pfap2-g* locus in the original individual parasite from which this subclone originated, because it was not possible to analyze heterochromatin until sufficient parasite biomass was obtained. We modified the text to acknowledge this limitation and indicate that the model proposed for the AP2-G-DD line is the most plausible explanation for the results obtained, but not fully demonstrated (**changes in lines 450 and 457-62**).

The experiment proposed by the Reviewer would be an interesting approach, but we see some problems with it: i) by deleting the region competent for heterochromatin nucleation we would also affect the expression and coding sequence of *pfap2-g*. Therefore, 100% conversion would not be achieved; ii) the AP2-G-DD line is not marker free (it carries an integrated *hdhfr* marker, see PMID 24572369, sup. fig. 6). This implies that further genetic editing is complicated, as it would require the use of an alternative marker, such as *bsd* (in our hands, the *bsd* marker does not work efficiently, as resistant parasites with the two *clag3* genes silenced are often selected); and iii) if the generation of correctly edited parasites was achieved, we would need to maintain cultures for weeks or months until parasites with and without heterochromatin spontaneously reached sufficiently high frequencies, and then isolate and analyze such subclones. Altogether, we estimate that the full experiment would take >6 months to perform, and it may provide limited new insight on heterochromatin biology. Given that none of the main conclusions of the manuscript is based on the analysis of the AP2-G-DD line, we preferred not to perform this complex and high-risk experiment. However, we modified the text to mention the limitations and improve the interpretation of the experiments with the AP2-G-DD line, as described above.

Reviewer #2: The study was somewhat affected by the difficulty of cloning the low complexity intergenic sequences and the uncertainty of their recombination. Overall I think the multiple experiments conducted control for these variables and extra experimentation to address them is not required although some analysis of their existing sequencing data might address concerns about off target integration or the presence of episomes/concatamers.

Line 242
“HC formation at the F0 and F1 fragments integrated in the genome is demonstrated by the detection of H3K9me3 with the external primer pairs 5’ext. and 3’ext., which recognize sequences adjacent to the site of integration that are not present in the transfection plasmids (Fig. 2A,C).”

This is true albeit the ext sequences are less enriched with H3K9me3 than the cloned sequence. How can the authors exclude the possibility of off target integration? It looks like F0 and F1 might be contained within the endogenous recombinant ap2g locus. Could there have been a low frequency of recombination here? The intergenic sequences could have recombined with other low complexity high %AT sequence in the genome but this doesn’t matter because they failed to nucleate heterochromatin.

This could be very difficult to resolve but a simple thing to try would be mapping the F1 input from fig4C (which has multiple integrated copies in fig 2D) to the various possible in silico modified recombinant loci. The authors could also filter for the inserted sequences and de novo assemble then map the assembled sequences flanking the cloned ap2g sequences back onto the genome to see whether the sequences might have integrated elsewhere.

We followed the suggestion from the Reviewer and used the ChIP-seq input data to assess if integration of the plasmid occurred at genomic loci other than the PF3D7_1144400 gene. We first used data from the 3E subclone (fig. 4A and 4F), which carries concatemer integration of the plasmid with the F1 fragment, as shown in Fig. 4B.

Our strategy was as follows:

1-First, we mapped ChIP-seq input reads against the plasmid used for the transfection (pL1144400-F1 plasmid). When one read mapped to the plasmid (mapping quality > 5) but its paired read did not (we used paired-end sequencing), the non-mapping read was retained.

2-Next, the non-mapping reads from the previous step were mapped against the wild type 3D7 genome (mapping quality > 5). The majority of reads mapped in two clusters at the PF3D7_1144400 locus, around the site of insertion. No reads mapped at the *pfap2-g* locus, and only a small number of isolated, scattered individual reads mapped elsewhere in the genome.

PF3D7_1144400 locus:

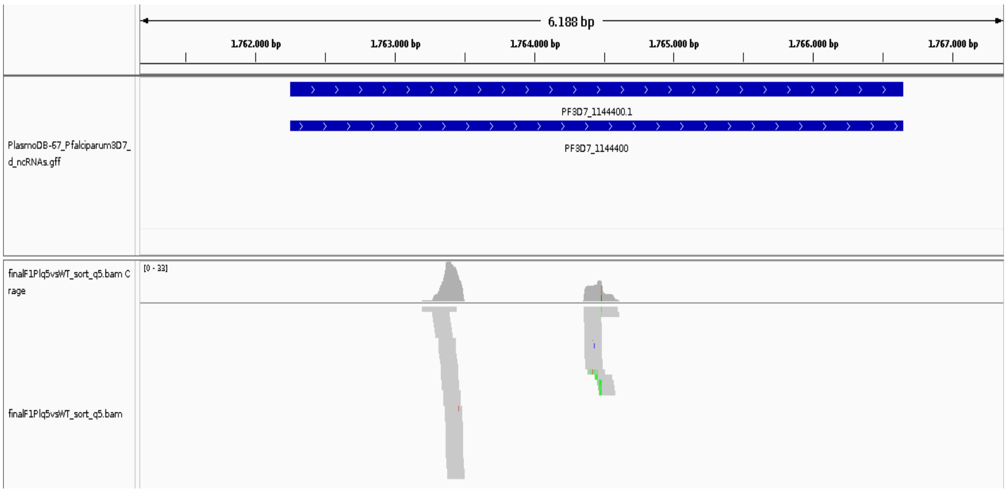

***pfap2-g* locus:**

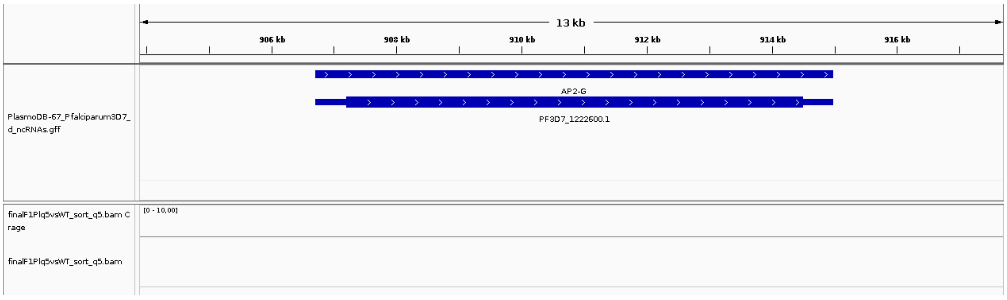

We preferred to use this unbiased approach rather than aligning reads against edited genomes with integration at all possible sites, because alignment against edited genomes would not exclude integration at unexpected sites via recombination with sequences similar to sequences present in the plasmid. Additionally, to test if there was integration at the *pfap2-g* locus, we could not simply align reads against edited genomes with the putative integration and check if there were reads spanning the integration junctions, because integration would occur by single homologous recombination and it would not generate junctions containing unique sequences.

We followed an analogous approach to assess integration of the F1+F0 fragment at genomic locations other than the PF3D7_1144400 locus in the A3 subclone. However, in this case a single copy of the fragment was integrated, without concatemer integration (Fig. 4D). Absence of plasmid backbone in the genome was validated by qPCR (**new graphs in Figs. 3E and 4D**). Therefore, for step 1 we mapped reads against the F1+F0 fragment, rather than against the full plasmid. The F1+F0 region was not completely deleted from the endogenous *pfap2-g* locus (base pairs 907,951-909,177 of chr. 12 were present in the endogenous *pfap2-g* locus and in the F1+F0 fragment integrated at the PF3D7_1144400 locus, highlighted in red in the figure below). For this reason, some reads from fragments arising from the endogenous gene were retained for step 2 and mapped to the *pfap2-g* locus at a position adjacent to the 907951-909177 region (these reads do not indicate integration at the *pfap2-g* locus). Alignment of the non-mapping reads against the 3D7 genome showed that essentially all reads mapped to the PF3D7_1144400 locus (except for those mapping to the above-mentioned fragment).

PF3D7_1144400 locus:

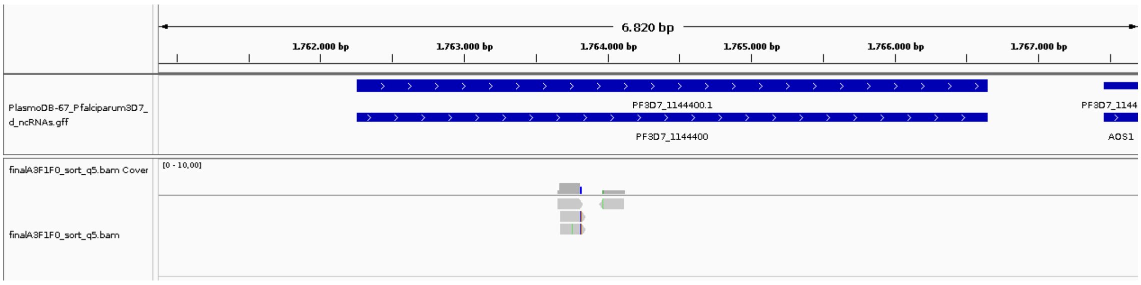

***pfap2-g* locus:**

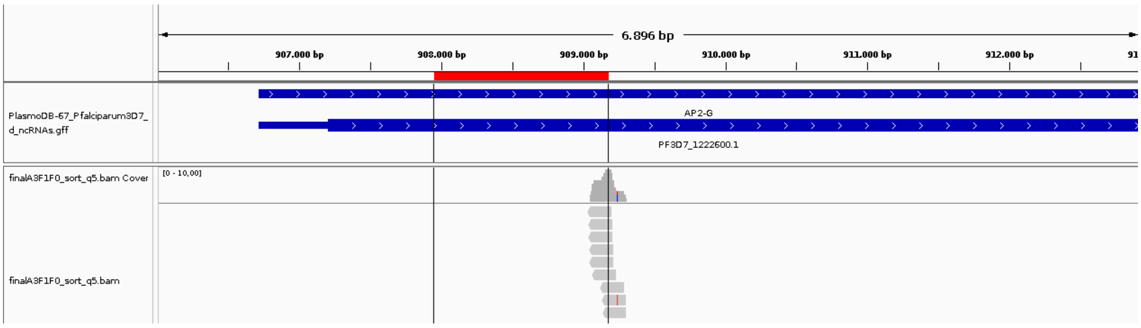

This analysis indicates that integration occurred at the desired locus. Furthermore, the parasite lines in which heterochromatin nucleation was confirmed by ChIP-seq are subclones, and therefore they are genetically homogeneous, excluding the possibility that integration may have occurred at other genomic sites in a fraction of the parasites.

We would also like to highlight that the region corresponding to the F1 fragment was deleted from the endogenous *pfap2-g* locus (see **modified Fig. 2B**, indicating the region deleted in the endogenous locus and the position of the fragments), making integration at this locus by homologous recombination with the F1 fragment in the plasmid impossible. Finally, in Fig. 2C, H3K9me3 coverage was lower at the “ext” positions than at the homology regions or the fragment itself, as pointed out by the Reviewer, but coverage at the “ext” positions was still clearly higher than in the negative control (euchromatic genes). The lower coverage at “ext” positions is expected because heterochromatin spreading is slow and typically requires multiple generations (see lines 115-9 and references therein). Signal at the “ext” regions was also clearly higher than in negative controls in the ChIP-qPCR analyses in Fig. 4A and C, and in the ChIP-seq experiments. This unambiguously indicates that heterochromatin was formed at the fragments integrated at the PF3D7_1144400 locus.

We preferred not to include the complex analysis of the ChIP-seq input sequences described above in the manuscript, but made the following changes to show that heterochromatin was formed in the fragments integrated at the PF3D7_1144400 locus:

-We indicate that H3K9me3 occupancy at the “ext” regions was clearly higher than in euchromatic ChIP-qPCR controls (**changes in lines 278-80 and 304-6**), which can only be explained by correct integration at the PF3D7_1144400 locus.

-We indicate that deletion of the sequences in the endogenous locus prevents integration at the *pfap2-g* locus (**changes in lines 228-9 and 241-3, and modified Fig. 2B**).

-We show that no plasmid backbone DNA could be detected by qPCR in the F1+F0 or F2+F1 lines (**new graphs in Fig. 3E and 4D**).

-In the Methods section, we explain that the analysis of ChIP-seq input sequences for lines F1 and F1+F0 fragments provided evidence for correct integration at the PF3D7_1144400 locus, including observation of reads mapping to the unique integration junctions containing hybrid PF3D7_114440 / *pfap2-g* sequences. Given that the lines analyzed were subclones and hence genetically homogeneous, this excludes the possibility that in a subset of the parasites the fragments were integrated elsewhere in the genome (**changes in lines 754-61**).

Illumina sequencing of the integration of the F1 fragment, with zoom in at the integration junction showing PF3D7_114440 / *pfap2-g* hybrid reads:

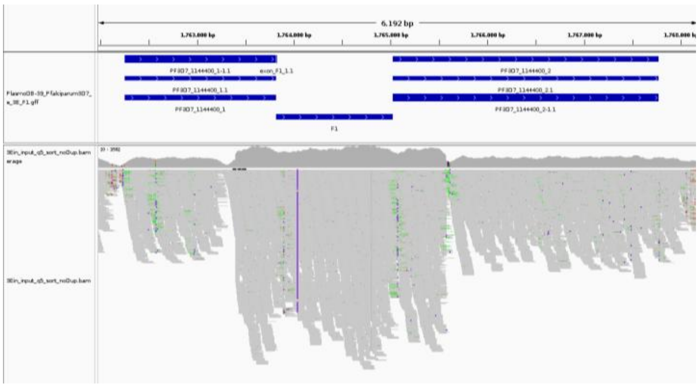

5' junction **3' junction**

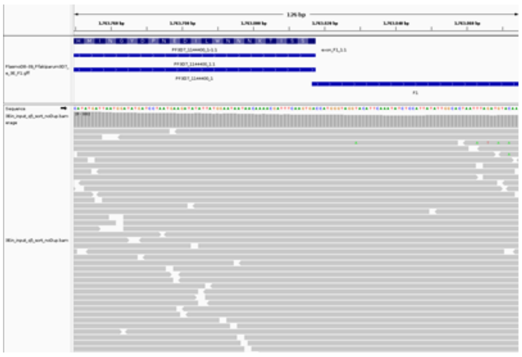

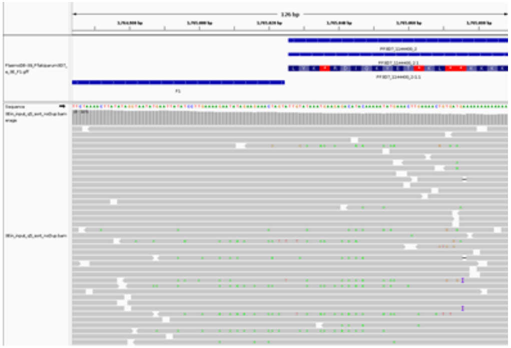

Reviewer #3: - The experiments integrating sequences of the AP2-G locus into the euchromatic Pf3D7_11444000 gene nicely delineate a sequence at the gene start to be capable of nucleating heterochromatin. However, the sequence is less efficient than parts of the 5' region of var genes. I was wondering: could the inefficient heterochromatin detection not also be a result of the absence of sequences from area -899 to -130, which were identified as critical for heterochromatin maintenance in Fig. 6 and 7? If I understand correctly, these sequences are missing in the clones carrying F1 or F1+F0.

This region is indeed missing from fragments F0 and F1+F0. However, fragment F2+F1 contains the -899 to -130 bp region and is unable to nucleate heterochromatin (Fig. 3D). Fragment F2 nucleates heterochromatin, but to a lower extent than fragment F1 or F0, and only when multiple copies are integrated (Fig. 2C-D). Therefore, we considered that this region, which as the Reviewer correctly points out is important for heterochromatin maintenance, does not appear to play a major role in nucleation.

To more clearly show how the relative position of the sequences that have nucleation capacity or are necessary for maintenance, we added a new panel to Fig. 7 showing the relative position of the regions important for heterochromatin nucleation or maintenance, relative to key landmark elements of the gene (**changes in lines 399-400, 480, and new Fig. 7B**).

- Sublcones were generated for U3', presumably because the original line still contained wt DNA according to Figure S10. As the clonal data for both, nucleation (F1+F0 or F1) and maintenance (U3') demonstrate a substantial degree of clonal variability, how confident are you that you don't miss an effect for the other (uncloned) regions?

Clonal variability was indeed a major difficulty for the identification of sequences able to nucleate heterochromatin or necessary for heterochromatin maintenance. However, for parasite lines with integrated fragments for which we did not obtain subclones, ChIP-qPCR values reflect the average H3K9me3 occupancy in the parasites population and the probability of the underlying sequence to become heterochromatic. For fragments in which there was no H3K9me3 signal above background, it is indeed possible that some individual parasites formed heterochromatin at the integrated fragment, but this would be a very low frequency event and identifying subclones in which heterochromatin was formed would require screening by ChIP-qPCR a very large number of subclones, which is not feasible.

We used the analysis of uncloned populations as a screening tool to identify the regions that have the capacity to nucleate heterochromatin at detectable levels. For selected regions that nucleated or maintained heterochromatin at intermediate levels, we generated subclones to characterize the resulting heterochromatin domains in more detail and to test if population heterogeneity was the source of intermediate levels. For integrated regions that did not form heterochromatin at detectable levels in uncloned populations, we considered that if they can nucleate heterochromatin they do it with very low efficiency. Likewise, for deleted regions that did not show a substantial reduction of heterochromatin at other parts of the locus, we considered that they do not play a major role in maintenance. To make this interpretation clearer, we modified the text of the Results (**changes in lines 260-4**).

- AP2-G is known to bind to its own promoter and enhance its own transcription. Are there particular sequence motifs evident in the areas for nucleation and maintenance (-899 to -130)? Where are the multiple AP2-G binding sites relative to the identified regions? Are any other predicted TF binding sites or motifs evident? In line 260, the authors mention sequence repetitiveness as a potential factor for making nucleation more effective. Therefore, I think analysis of the primary sequence may help in understanding the results better.

We modified several figures to indicate the position of the PfAP2-G binding sites at the *pfap2-g* upstream region (**new Fig. 1, 2B, 3C, 6B and 7B**) and performed an analysis of the predicted binding sites for other ApiAP2 transcription factors at the *pfap2-g* regions able to nucleate heterochromatin or needed for maintenance.

PfAP2-G binding sites do not overlap with the fragments important for either nucleation or maintenance, which is now stated in the text (**changes in lines 482-5**) and clearly shown in **new Fig. 7B**. Regarding other transcription factors, we performed an analysis of the experimentally mapped binding sites for several ApiAP2s (PMID 34365503) and the presence of the cognate binding sites for most ApiAP2s (PMID 21060817) at the *pfap2-g* locus. The results showed that multiple ApiAP2s, including AP2-EXP, AP2-P, AP2-O2, AP2-12 or AP2-G5 likely bind within the regions important for heterochromatin nucleation or maintenance, but these factors have a broad distribution in the genome, with multiple binding sites across the *pfap2-g* locus and across the full genome. Since this analysis did not identify any specific candidate factor that may be involved in heterochromatin nucleation or maintenance, we considered that it was not informative and preferred not to include it in the manuscript. However, we added a sentence in the Discussion regarding the results of this analysis (**changes in lines 485-8**).

We also analyzed the presence of repetitive sequences at the *pfap2-g* regions needed for maintenance or nucleation. To assess if these regions contained long repeated regions, beyond simple short nucleotide repeats (typically mono- or di-nucleotides), which are present in essentially all intergenic and many protein coding regions, we generated dot plots of the region from -2,000 to +2,000 bp from the start codon (ATG) against itself. Repeated regions are apparent as lines parallel to the main diagonal. This analysis did not reveal any long repetitive region at the *pfap2-g* locus, in contrast to the *eba175* locus that contains a ~450 bp repeated region and was used as a positive control.

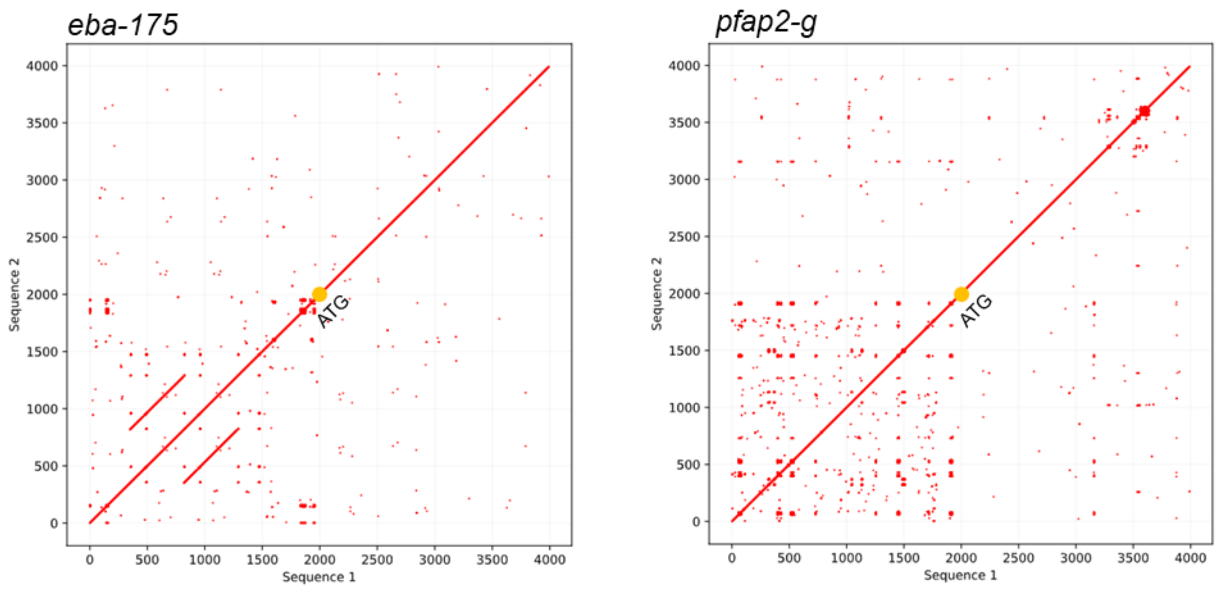

Since the result of this analysis was negative, we preferred not to include it in the manuscript.

Part III – Minor Issues: Editorial and Data Presentation Modifications
Please use this section for editorial suggestions as well as relatively minor modifications of existing data that would enhance clarity.

Reviewer #1:

• Fig S2: It is unclear what the difference is between the left and right graphs. Are they for different clones or replicates?

We apologize for the lack of clarity for S2 Fig. in the previous version of the manuscript. All data correspond to the Δ5'ap2-g line (subclone H11). In the revised manuscript, we modified the legend of this figure to explain that the two panels correspond to separate ChIP-qPCR experiments in which different genes were analyzed (although some genes were included in both experiments).

• Fig. 2C: I always prefer to see raw values rather than ratios. Maybe the authors could put bar graphs in supplementary data with raw values for each primer pair used, including separate bars for each heterochromatic and euchromatic control locus? This is just to see the variability amongst the different loci for one experiment, not necessary for the rest of the manuscript. Since they only have two replicates, I think it would be more transparent to provide each data point on the bar graph rather than have error bars.

Following the Reviewer's suggestion, **we added a new panel in S2 Fig. (panel C)** with the raw (% of input recovered) results from the ChIP-qPCR experiments in main Fig 2C. The dispersion of the data is larger, as expected, because the absolute recovery from the IP is different between different experiments. However, in this new raw data graph it is still very clear that fragments F0 and F1, and to a lesser extent F2, induce

heterochromatin formation at levels above euchromatic controls, whereas for other fragments H3K9me3 levels are undistinguishable from euchromatic controls.

We also **modified all figures presenting bar graphs** to display individual data points, as suggested by the Reviewer.

• Could the authors please provide a figure (or just add to Fig. 2B) showing H3K9me3 and/or HP1 ChIP-seq data (already published is fine) enrichment over the ap2-g and 1240300 loci? Just to give a visual of the normal chromatin composition of the fragments tested.

Following the Reviewer's suggestion, we **modified Fig. 2B** and also **Fig. 5A,C** to show the distribution of heterochromatin in the parental wild type 1.2B line, from a previous study that used ChIP-seq.

• Have the authors tested the primer efficiencies used for ChIP-qPCR to confirm that the different levels of enrichment aren't due to differences in AT-richness. For example, regions up- and downstream of the HR/internal Fx sequences might be less GC rich and lead to less relative amplification.

For all qPCR experiments in this manuscript, we used the standard curve method (ref. 68, PMID: 34313989), in which a standard curve (made with parasite gDNA) is included in each plate for each primer pair. With this method, relative quantity for each gene in the sample cDNAs is calculated by interpolating Ct values against the standard curve. Since primer efficiency is the same in the standard curve and in the sample, results are largely insensitive to primer efficiency. We modified the Methods section to clarify this important aspect of our ChIP-qPCR analysis (**changes in lines 658-9 and 822-3**). In any case, all primers used for qPCR had an efficiency >80%, which is now also stated in the Methods (**changes in lines 658-9 and 822-3**).

• Fig. 4E/F: It's interesting that H3K9me3 coverage tapers off just at the start and end codons of the 1144400 gene. The authors discuss how barrier elements are well defined for normal heterochromatin domains, but could the authors comment on why they think HC doesn't spread into the upstream/downstream regions of this euchromatic gene?

We indeed found it intriguing that heterochromatin spreading stopped around the start and stop codons of the gene PF3D7_1144400, located in an euchromatic region. For a gene that is not usually near heterochromatin domains, barrier elements would not be expected. We do not have a satisfactory explanation for the heterochromatin limits at this locus, but as an alternative explanation we speculate that parasites in which heterochromatin spreads into the intergenic regions may be selected against, because heterochromatin would interfere with the expression of the neighbor genes (**changes in lines 571-7**). Of note, these genes encode likely essential proteins, a ribosomal protein and a SUMO enzyme subunit.

• Could the authors please provide western blots of AP2-G-DD for the different subclones in Fig. 8 (with and without shield) to show that the differences in H3K9me3 levels at the ap2-g locus correspond to different levels of AP2-G expression?

We performed new experiments to confirm that *ap2-g* expression levels are indeed different between the two subclones. We preferred to assess *ap2-g* transcript levels (by RT-qPCR) rather than AP2-G protein levels, because in the absence of Shld1, AP2-G is degraded and its levels cannot be determined by Western blot. Since

heterochromatin levels in panels D-E were measured in the absence of Shld1, we considered that it is more appropriate to measure *ap2-g* transcript levels, because they can be measured both in the presence and in the absence of Shld1.

Using ML10-treated cultures at the late schizont stage (to prevent stage differences between the subclones), we found that *ap2-g* expression was >50-fold higher in the B11 subclone. The results of this experiment are presented in a **new panel of Fig. 8 (new panel C)** and described in the text **(changes in lines 433-5**). We also modified the Methods **(changes in lines 624-8**).

Reviewer #2:

Line 259

"Formation of HC only in parasite lines with multiple copies of F0 or F1 may be explained by the larger total size of putative HC-promoting DNA or by sequence repetitiveness (occurrence of the same sequence more than one time), as repetitiveness itself may promote HC formation. In Drosophila and other organisms, presence of multiple copies of a transgene is sufficient to trigger HC-based silencing [48, 49]. To distinguish between these possibilities"

Another possibility is off target integration into a heterochromatic locus. I couldnt see a plasmid map of the pDC2 plasmid and the earliest citation I could find DOI: 10.1038/NMICROBIOL.2016.166 didn't have a plasmid map. Does the plasmid contain other P falciparum sequences that could mistarget the integration?

Please note that plasmid pDC2 carries the sgRNA, Cas9 and hdhfr/yfcu expression cassettes (S4 and S9 Fig.), but it is not intended for integration in the genome or for testing heterochromatin nucleation (it does not carry fragments from the *pfap2-g* or any other heterochromatic locus). The plasmids used to integrate fragments to test heterochromatin nucleation were pL1144400-Fx (S4 Fig.) and pL1144400-Fx-yFCU (S9 Fig.).

Plasmid pL1144400-Fx only carries the fragment of interest, the PF3D7_1144400 homology regions and *E. coli* sequences (S4 Fig.). The pL1144400-Fx-yFCU plasmid also carries the yFCU cassette, which includes the *P. falciparum hsp90* promoter (the terminator is a *P. berghei* sequence, not from *P. falciparum*) but no heterochromatic sequence that could drive integration into heterochromatic parts of the genome (S9 Fig.). We consider that integration at heterochromatic sites of the genome cannot explain the formation of heterochromatin observed with the F0, F1 or F1+F0 fragments for the following reasons:

-For transgenic lines with integrated fragments for which we performed ChIP-seq analysis, analysis of input sequences did not reveal unwanted integration at other sites of the genome, and they are subclones (genetically homogeneous). Please see the response to the Major Comment from Reviewer 2 for details.

-We clearly observed heterochromatin signal at the PF3D7_1144400 locus at positions external to the homology regions ("ext" primers), which cannot be explained by integration at heterochromatic regions.

Therefore, we preferred not to add the third possibility pointed out by the Reviewer because we consider it highly unlikely. However, we made all the changes in the manuscript described in response to the Major Comment from Reviewer 2 to make clear that the heterochromatin observed does not arise from integration elsewhere in the genome.

Line 288

“These results confirm that the intermediate levels of H3K9me3 observed in transgenic lines with HC-positive pfap2-g fragments reflect population heterogeneity rather than partial H3K9me3 occupancy in individual parasites”.
I don’t think the data excludes the possibility that clones can have differing levels of H3K9me3 at a locus, the two heterochromatic ap2g-f1 subclones have differing levels of heterochromatin and the individual clones have different levels at the different pcr sites.

We agree with the Reviewer that while our data clearly demonstrates population heterogeneity (shown by differences between subclones), it does not fully exclude the possibility of partial H3K9me3 occupancy. We modified the text, deleting the last part of the sentence (**changes in line 326**).

Line 388
“ChIP-seq analysis of cultures maintained without Shld1 revealed a lower H3K9me3 coverage at the pfap2-g locus in the parental E5-AP2-G-DD line and a subclone with high sexual conversion, compared with a subclone with low conversion. This is consistent with the gene being in an euchromatic state in the approximately 50% of parasites that convert upon Shld1 addition (Fig. 8C-E).”
This statement requires some statistical support from the chipseq data, e.g. diffbind.

Unfortunately, we could not perform diffbind analysis or any other differential peak calling analysis to obtain statistical support for this result, because these methods require biological replicates. However, in the revised manuscript we provide a statistical analysis using a permutation test, which shows that the different heterochromatin coverage at the *pfap2-g* locus between D7 and B11 was statistically significant (**changes in lines 440-4 and 856-61**).

We also provide a new Supplementary Dataset (**new S1 Dataset**) in which it is apparent that the difference observed at the *pfap2-g* locus is among the top differences in the full genome (14th largest fold-change for the D7 vs B11 H3K9me3 coverage comparison). We modified the text to cite the **new S1 Dataset** and to explain these results (**changes in lines 440-4**). Only a few genes showed higher or similar levels of H3K9me3 coverage fold-change between D7 and B11, mainly genes involved in antigenic variation (*var*, *rif*, *pfmc-2tm*, …), known to show marked heterochromatin and expression variation between subclones (PMID 36515553).

Line 420
“Of note, the regions needed to maintain pre-existing HC are different from those capable to nucleate HC de novo”
This is difficult to assess from the presented data. Perhaps a figure showing which bits were tested for nucleation and were effective (F1, F0) and which were required for maintenance (deltaU, deltaU3'-in some subclones) depicted on the same diagram would help. As it stands it seems that delta U3' and F0 and F1 overlap.

Following this excellent suggestion from the Reviewer, we prepared a new panel for Fig. 7 (**new Fig. 7B**) showing the relative position of the regions important for heterochromatin nucleation and maintenance, together with other landmark elements. This new figure, cited several times in the text, clearly shows that there is only a minor overlap between the U3' and the F1+F0 fragments.

Reviewer #3:

- Line 191: to control for uneven amplification due to AT bias for D1 and D2 fragments, the authors should use a gDNA titration to determine the primer efficiency and account for this in their calculation (Pfaffl method). This should help to distinguish between technical limitations and true biological variation**.**

As explained in the response to the fourth minor comment from Reviewer 1, for all qPCR experiments in this manuscript we used the standard curve method (ref. 68, PMID: 34313989), in which a standard curve is included in each plate for each primer pair. Relative quantity for each gene in the sample cDNAs is calculated by interpolating against the standard curve. Since primer efficiency is the same in the standard curve and in the sample, results are largely insensitive to primer efficiency. We modified the Methods section to clarify this important aspect of our analysis (**changes in lines 658-9 and 822-3**).

- Fig. S2 is cited as a reference in line 224 – 227 for cloned var and ama1 loci, but the figure only gives information of endogenous HC levels of these genes. I think this is shown in Fig. 2B.

We cited S2 Fig. in this sentence to show that the endogenous *var* and *ama1* genes are indeed heterochromatic and euchromatic, respectively, in the parasite line used for fragment integration. We agree that the way it was phrased may be confusing, and consequently we modified the text to make this clearer (**changes in lines 254-7 and also 219-20**).

- The experiments integrating sequences of the AP2-G locus into the euchromatic Pf3D7_11444000 gene nicely delineate a sequence at the gene start to be capable of nucleating heterochromatin. However, the sequence is less efficient than parts of the 5' region of var genes. I was wondering: could the inefficient heterochromatin detection not also be a result of the absence of sequences from area -899 to -130, which were identified as critical for heterochromatin maintenance in Fig. 6 and 7? If I understand correctly, these sequences are missing in the clones carrying F1 or F1+F0.

Please note that this comment is repeated and it was already answered in the Major Comments section.

- Sublcones were generated for U3', presumably because the original line still contained wt DNA according to Figure S10. As the clonal data for both, nucleation (F1+F0 or F1) and maintenance (U3') demonstrate a substantial degree of clonal variability, how confident are you that you don't miss an effect for the other (uncloned) regions?

Please note that this comment is repeated and it was already answered in the Major Comments section.

- Fig. 4F indicates that the heterochromatin boundaries are strictly delimited by the CDS of Pf3D7_1144400. Could the authors speculate what may determine those boundaries in this locus?

Please see the response to Minor comment 5 from Reviewer 1, where we speculate about this intriguing observation and describe the changes done in the text (**changes in lines 571-7**).

- In Fig 4E the H3K9ac pattern is shown in the recombinant locus. What is the H3K9ac/H3K9me3 pattern in WT parasites at this locus? Is this gene normally transcribed and carries promoter acetylations (though the gene is non-essential)?

We thank the Reviewer for this suggestion. We agree that it is important to show the distribution of the histone modifications at this locus in wild type parasites. We added a **new panel to Fig. 4 (new panel 4E)** including H3K9me3 and H3K9ac ChIP-seq data for the wild type parental line (1.2B) from a previously published study by our team (ref. 16, PMID 36515553).

- In Fig 4E the H3K9ac pattern is shown in the recombinant locus. What is the H3K9ac/H3K9me3 pattern in WT parasites at this locus? Is this gene normally transcribed and carries promoter acetylations (though the gene is non-essential)?

Please note that this comment is repeated and it was already answered above.

- AP2-G is known to bind to its own promoter and enhance its own transcription. Are there particular sequence motifs evident in the areas for nucleation and maintenance (-899 to -130)? Where are the multiple AP2-G binding sites relative to the identified regions? Are any other predicted TF binding sites or motifs evident? In line 260, the authors mention sequence repetitiveness as a potential factor for making nucleation more effective. Therefore, I think analysis of the primary sequence may help in understanding the results better.

Please note that this comment is repeated and it was already answered in the Major Comments section.

- What is the hypothesis on heterochromatin re-established once the parasites have entered the sexual differentiation program? AP2-G is only briefly unpacked and expressed during commitment and regains a heterochromatic state during gametocyte differentiation. Wouldn't this argue against the inheritance model of heterochromatin nucleation or do the authors think that other mechanisms are relevant in this context?

Data from us and from others suggest that activation of *pfap2-g* during sexual commitment and early gametocyte development is associated with retraction of heterochromatin at the promoter region, rather than complete dismantling of heterochromatin at the full locus (PMID 39369041 and 37013531). Therefore, reestablishment of heterochromatin after commitment may occur by expansion from the coding sequence and not require *de novo* nucleation. We modified the text to include this information, with references (**changes in lines 538-40**).

- As var genes are grouped into subgroups (A-E) according to their 5' regions and genomic position that differ significantly in their switching dynamics, it would be meaningful and informative to report what group the analysed variants belong to.

We added the information for *var* groups (**changes in lines 345-7**).

- Please use parasite line names consistently throughout all figures, e.g. I believe the line shown in Fig. 6A as Δ5'ap2-g (F1+F0 sc A3) is referred to as A3_F1+F0 in Fig. 4 with the heading "ap2-g F1+F0 subclones". I found this at times hard to follow. A family tree of the clones may be helpful.

We thank the Reviewer for identifying this mistake. **We modified Fig. 6A** to use the same nomenclature as in Fig. 4. However, after the name of the line, we added in brackets "(Δ5'ap2-g)", because in Fig. 6A, centered on the *pfap2-g* locus, we consider it relevant to indicate how this locus was edited (previous to integration of the fragment).

As suggested by the Reviewer, **we also added a family tree of the different parasite lines to S2 Fig. (new panel B) and refer to it in the text** (**change in lines 236**). We hope this will help to make the manuscript easier to follow.

- Please report SD, not SEM for all graphs. SEM is an unsuitable statistical value for an experiment with two replicates.

We changed the error bars in all the graphs (main and supplementary figures) to show S.D. instead of S.E.M., and updated the figure legends accordingly.

- Please mention why the expected copy number level in Fig. 2D higher (2x) for ama1 and Pf3D7_1240300. Is this because the endogenous sequence has not been deleted?

The Reviewer is correct. We added this explanation in the legend of Fig. 2D (**changes in lines 1,170-73**).

- Please indicate the location of the TSS of the genes in Fig. 5A, C

In a published genome-wide study specifically focused on TSS mapping, using a modified version of the RNA-seq-based CAGE method (PMID 26947071), the TSSs for *mspdbl2* or the *var* genes were not mapped. Therefore, we could not indicate the position of the TSSs for these genes. The most plausible reason for not detecting TSSs in the promoters of these genes is that they are silenced in the vast majority of parasites, precluding the occurrence of sufficiently high numbers of transcripts for TSS identification.

Other studies, using DAFT-seq (PMID 32513207) or RNA-seq enriched for the 5' end of complete transcripts (PMID 26578577), reported putative TSSs for some of the genes, but these were almost adjacent to the start codon and occurred at levels similar to putative TSSs in the coding sequence (unlikely to be authentic TSSs). Therefore, we considered that those were possibly low confidence TSSs and preferred not to report them.

- In Fig. 7, I would suggest to highlight the identified "heterochromatin maintenance" region between-899 to -130.

We added a new panel to Fig. 7 (new panel 7B) showing the position of the regions with important roles for heterochromatin nucleation or maintenance at the *pfap2-g* locus.

- Fig. 4F indicates that the heterochromatin boundaries are strictly delimited by the CDS of Pf3D7_1144400. Could the authors speculate what may determine those boundaries in this locus?

Please note that this comment is repeated and it was already answered above.

- What is the hypothesis on heterochromatin re-established once the parasites have entered the sexual differentiation program? AP2-G is only briefly unpacked and expressed during commitment and regains a heterochromatic state during gametocyte differentiation. Wouldn't this argue against the inheritance model of heterochromatin nucleation or do the authors think that other mechanisms are relevant in this context?

Please note that this comment is repeated and it was already answered above.
